# Single-cell and bulk RNA sequencing reveal ligands and receptors associated with worse overall survival in serous ovarian cancer

**DOI:** 10.1186/s12964-022-00991-4

**Published:** 2022-11-09

**Authors:** Robson Francisco Carvalho, Luisa Matos do Canto, Cecilie Abildgaard, Mads Malik Aagaard, Monica Søgaard Tronhjem, Marianne Waldstrøm, Lars Henrik Jensen, Karina Dahl Steffensen, Silvia Regina Rogatto

**Affiliations:** 1 Department of Clinical Genetics, University Hospital of Southern Denmark, 7100 Vejle, Denmark; 2 Institute of Regional Health Research, University of Southern Denmark, 5230 Odense, Denmark; 3 Department of Structural and Functional Biology - Institute of Bioscience, São Paulo State University (UNESP), 18.618-689, Botucatu, Brazil; 4 Department of Oncology, University Hospital of Southern Denmark, 7100 Vejle, Denmark; 5 Danish Colorectal Cancer Center South, 7100 Vejle, Denmark; 6 Department of Clinical Pathology, University Hospital of Southern Denmark, 7100 Vejle, Denmark

**Keywords:** Cancer-associated fibroblasts, Organoids, Serous ovarian cancer, Single-cell RNA-sequencing, Cell–cell communication

## Abstract

**Background:**

Serous ovarian carcinoma is the most frequent histological subgroup of ovarian cancer and the leading cause of death among gynecologic tumors. The tumor microenvironment and cancer-associated fibroblasts (CAFs) have a critical role in the origin and progression of cancer. We comprehensively characterized the crosstalk between CAFs and ovarian cancer cells from malignant fluids to identify specific ligands and receptors mediating intercellular communications and disrupted pathways related to prognosis and therapy response.

**Methods:**

Malignant fluids of serous ovarian cancer, including tumor-derived organoids, CAFs-enriched (eCAFs), and malignant effusion cells (no cultured) paired with normal ovarian tissues, were explored by RNA-sequencing. These data were integrated with single-cell RNA-sequencing data of ascites from ovarian cancer patients. The most relevant ligand and receptor interactions were used to identify differentially expressed genes with prognostic values in ovarian cancer.

**Results:**

CAF ligands and epithelial cancer cell receptors were enriched for PI3K-AKT, focal adhesion, and epithelial-mesenchymal transition signaling pathways. Collagens, MIF, MDK, APP, and laminin were detected as the most significant signaling, and the top ligand-receptor interactions THBS2/THBS3 (CAFs)—CD47 (cancer cells), MDK (CAFs)—NCL/SDC2/SDC4 (cancer cells) as potential therapeutic targets. Interestingly, 34 genes encoding receptors and ligands of the PI3K pathway were associated with the outcome, response to treatment, and overall survival in ovarian cancer. Up-regulated genes from this list consistently predicted a worse overall survival (hazard ratio > 1.0 and log-rank *P* < 0.05) in two independent validation cohorts.

**Conclusions:**

This study describes critical signaling pathways, ligands, and receptors involved in the communication between CAFs and cancer cells that have prognostic and therapeutic significance in ovarian cancer.

**Video abstract**

**Supplementary Information:**

The online version contains supplementary material available at 10.1186/s12964-022-00991-4.

## Background

The most common histology of malignancies of the ovary is epithelial cancer, recognized by the World Health Organization as low- and high-grade serous ovarian cancer [1, 2]. High-grade serous ovarian cancer (HGSOC) is the most prevalent and aggressive form of the disease and the leading cause of ovarian cancer deaths [1, 3]. The main therapeutic strategies are debulking surgery and platinum-based chemotherapy [4, 5]. The cytoreductive surgery is performed as primary treatment or as interval debulking surgery in conjunction with neoadjuvant chemotherapy [6]. The asymptomatic nature of ovarian cancer leads to an advanced diagnosis (FIGO stage III-IV) in 70% of cases, which results in a poor prognosis of ~ 25% 5-year survival for patients with stage IIIC-IV disease [7]. Furthermore, more than 30% of patients at advanced stages of the disease have malignant ascites or pleural effusions at diagnosis and are associated with recurrence and metastasis [8–10]. These fluids are drained regularly to relieve pain and discomfort and represent an inexpensive and less invasive source of metastatic tumor cells [11].

Communication and interaction between cancer cells and cancer-associated fibroblasts (CAFs) facilitate metastasis development and tumor progression [12, 13]. CAFs play a crucial role in these processes by producing components of the extracellular matrix (ECM) and secreted factors, which also influence angiogenesis, immunity, remodeling of the ECM, chemoresistance, and response to treatment (reviewed in [13, 14]). However, a comprehensive evaluation of the interactions between CAFs and cancer cells from malignant effusion fluids has not yet been determined.

Single-cell RNA sequencing (scRNA-Seq) has provided new insights into cancer biology by revealing the landscape of intercellular interactions and cell communication and improving the knowledge of tumor heterogeneity [15, 16]. In HGSOC, scRNA-Seq has been used to determine the transcriptomic heterogeneity of cancer and stromal cells from ascites and tumor tissues with a high-resolution [17–20]. Using scRNA-Seq in HGSOC, Kan et al. reported that CAFs induce epithelial-mesenchymal transition (EMT) of tumor cells via TGFβ signaling, with consequent effects on chemoresistance and metastasis [21]. A previous study showed that changes in the ECM promote an adaptive response during the co-evolution of stromal and cancer cells that drive aggressiveness in HGSOC patients [22].

In this study, we analyzed intercellular communication networks in malignant fluids from ovarian cancer patients. The inference and analysis of cell–cell communication and the signature of CAFs were obtained from the scRNA-Seq data [17] and combined with our RNA-Sequencing (RNA-Seq) data. We demonstrated that ligands and receptors of the PI3K-AKT pathway are essential mediators of CAFs communication with cancer cells. A set of these ligands and receptors genes was associated with an unfavorable prognosis. We also described promising targets for therapy.

## Materials and methods

### Patient samples and data collection

Patients diagnosed with serous ovarian cancer referred to the University Hospital of Southern Denmark, Vejle, DK, between February 2020 and March 2021 to drain ascites or pleural effusion were eligible to participate in the study. All patients signed an informed consent form. Malignant effusion fluids were drained and transferred to 50 mL tubes for further analysis. We also included eight normal ovarian tissue samples obtained from individuals who underwent surgery for other causes than cancer. Each sample was coded, and the personal health information was removed. Clinical and histopathological data were provided in a de-identified manner. Clinical data were obtained from patient records (Additional file 14: Table S1).

### Sample handling and cell culture

Malignant effusion fluids (four ascites and four pleural effusions) were immediately processed after drainage. Fluids were centrifuged (1000 rpm/5 min), and the pellet was treated with AKC lysis buffer (Merck, Darmstadt, Germany) for 5 min at room temperature to remove erythrocytes. The pellet was resuspended DMEM/F12, GlutaMAX (Gibco, Thermo Fisher Scientific, Waltham, USA) supplemented with 10% fetal bovine serum (FBS; Biological Industries, Beit HaEmek, Israel) and 1% antibiotic–antimycotic 100x (Gibco, Thermo Fisher Scientific, Waltham, USA). Cell number, live-cell fraction, and cell size distribution were determined using the Countess II Automated Cell Counter (Thermo Fisher, Waltham, USA). Ten mL of media were used to incubate the cells during the first 12 h at 37^o^ C with 5% CO2. After 12 h, cells attached to the flasks were cultured in two-dimensional (2D), and the supernatant was collected and centrifuged (1000 rpm/5 min) for three-dimensional (3D) cell culture. The cells were washed in medium and resuspended on ice in (1:1) cold StemPro™ hESC SFM growth full medium (which is composed by DMEM/F-12, GlutaMAX™ medium bovine serum albumin 25%, StemPro® hESC Supplement) (Thermo Fisher Scientific, Waltham, USA), added with FGF (10 µg/mL) (Thermo Fisher Scientific, Waltham, USA), 2-Mercaptoethanol (Thermo Fisher Scientific, Waltham, USA), Penicillin/Streptomycin (10,000U/ 10 mg/mL), Gentamycin, Amphotericin (2.5 µg/mL each) (Sigma-Aldrich, St. Louis, MO, USA), and Matrigel® Basement Membrane Matrix (1: 1full medium; Corning, New York, USA). We used 75 µL of the full media mixture in each well of a 24-well plate and kept it for 30 min at 37 °C, 5% CO2. After incubation, 1 mL of medium and supplements were added to each well, and the cells were incubated at 37 °C, 5% CO2. The medium was changed every 2–3 days, and 3D cells were harvested using Cell Recovery Solution (Corning, New York, USA), according to the manufacturer's protocol. Two-dimensional cell culture when confluent (5 to 10 days) was harvested with 0.5% trypsin–EDTA (Gibco, Thermo Fisher Scientific, Waltham, USA) and pellets were used for RNA isolation. Tumor-derived organoids (TDO) developed within 1–3 weeks.

### Histological characterization

Primary tumor tissues, uncultured cells on day 0 (baseline), and TDO (6 to 15 days) were fixed and processed for histological analysis using automated protocols (Tissue-Teck VIP 6AI Tissue Processor, Sakura Finetek, Japan). Representative sections of primary tumors and TDO were stained with hematoxylin and eosin (H&E) and compared by a board-certified pathologist. The expression of CK7, TP53, PAX8, and calretinin was evaluated by immunohistochemistry assays in primary tumors and their derived organoids using the Benchmark Ultra automated instrument (Ventana Medical Systems, Roche, Tucson, USA) or Dako Omnis (Agilent, Santa Clara, USA) (Additional file 14: Table S2).

### RNA extraction and sequencing analysis

The RNeasy mini kit (Qiagen, Valencia, CA, USA) was used to isolate total RNA from all samples (malignant effusion cells or baseline, 2D cells, TDO, and normal ovarian tissues) following the manufacturer's recommendations. RNA purity and quantity (Nanodrop spectrophotometer, Thermo Fisher Scientific, Waltham, USA) and its integrity (RNA screen tape on a 2200 TapeStation, Agilent, Santa Clara, USA) were evaluated before downstream applications.

Total RNA (100 ng) was used to prepare libraries with the Illumina Stranded Total RNA Prep Ligation with Ribo-Zero Plus kit (Illumina, San Diego, California, USA), following the manufacturer's protocol. TruSeq RNA UD indexes (Integrated DNA Technology – IDT, Coralville, Iowa, USA) were added to multiplex the samples, which were paired-end sequenced on the NovaSeq 6000 system (Illumina) using the S4 Reagent Kit v1.5 (300 cycles), according to the supplier's recommendations.

RNA-Seq data quality control was performed using FastQC and MultiQC [23, 24]. The reads were aligned with the Ensembl human genome assembly GRCh38 (release 99) using STAR (v.2.7.6a) [25], and the expression count matrix was generated using HTSeq [26]. The principal component plot and heatmaps were generated using the Galaxy [27] or Morpheus [28] platforms on log-transformed DESeq2-normalized counts [29]. Cluster analyses based on Euclidean distance, variance, or marker selection were also performed using Morpheus [28]. We selected the top 1,000 highly variable genes (highest standard deviation) across all samples to identify markers for malignant effusion cells, 2D cells, TDO, and normal ovarian tissues. The highly expressed genes in 2D cell cultures were inputted into the EnrichR tool [30–32] for a gene set enrichment analysis using the WikiPathways 2021, MSigDB Hallmark 2020, and Biological Process 2021 (GO) libraries. The top five enrichment terms (lowest adjusted *P*-value, Fisher's exact test) for each library were included in a final consensus list. The enrichment terms were represented in heatmaps generated by the web tool Morpheus [28].

### Single-cell RNA-sequencing data processing

Droplet-based scRNA-Seq data for eight HGSOC ascites samples were downloaded from Gene Expression Omnibus[33] (GSE146026). The identification and annotation of the 9609 cells to 18 cell clusters was obtained from the original publication [17] and processed using the default parameters of Seurat V3 [34]. Cells were clustered and visualized using the t-distributed stochastic neighbor embedding (tSNE) or violin plots in Seurat. The differentially expressed genes (DEGs, positive and negative cluster markers) were defined with the 'FindAllMarkers' Seurat function. We subset the cells into four clusters containing myofibroblastic and inflammatory CAFs. This subset was then used to identify the markers among the fibroblasts clusters and visualized using the tSNE or violin plots in Seurat. The list of DEGs for each cluster was inputted into the EnrichR tool [30–32] for a gene set enrichment analysis using WikiPathways 2021 and MSigDB Hallmark 2020. As described above, we included the top five enrichment terms (lowest adjusted *P*-value, Fisher's exact test) for these libraries in a final consensus list. The heatmaps for these enrichment terms were generated with the web tool Morpheus [28]. The expression profile of 40 CAFs markers (Top 10 from each CAF cluster) was analysed in our ovarian bulk RNA-Seq data to identify samples enriched with cells expressing CAF marker genes.

### Analysis of cell–cell communication

We quantitatively inferred and analyzed cellular communication networks from the HGSOC scRNA-seq data using CellChat [35] (v.1.1.3). To identify the potential ligand-receptor interactions, we loaded the Seurat object into CellChat (https://github.com/sqjin/CellChat/; accessed on 01/10/2021) using the default parameters. This analysis included 1939 human-validated interactions for paracrine/autocrine signaling (61.8%), extracellular matrix receptor interactions (ECM) (21.7%), and cell–cell contact interactions (16.5%). The aggregated cell–cell communication network was calculated by counting the interactions (represented in a circle plot), followed by the identification of cell–cell communication mediated by significant interactions (ligand-receptor pairs represented in bubble plots). We also filtered and explored specific interactions between CAFs and epithelial cancer cells using the webtool Morpheus [28]. Finally, CellChat was used to identify global communication patterns among cell types. The Cophenetic and Silhouette metrics were initially used to determine the number of incoming and outgoing communication patterns between cell groups and pathways. The signals that contribute the most to outgoing or incoming communication signaling (probability score) for each cell group were then identified and visualized using heatmaps and bar graphs.

### The Cancer Genome Atlas (TCGA) and Genotype-Tissue Expression (GTEx) RNA-sequencing data analysis

The expression levels [log2(normalized counts + 1)] of selected ligands and receptors genes (PI3K-AKT signaling pathway) in serous ovarian cancer (The Cancer Genome Atlas, TCGA, *n* = 418) and healthy tissues (Genotype-Tissue Expression, GTEx, *n* = 88) were downloaded from UCSC Xena [36] (https://xenabrowser.net/). The ligands and receptors genes for PI3K-AKT signaling were selected for further analysis based on consistent findings of the communication patterns between CAFs and cancer cells. The DEGs were identified by Welch's *t*-test and presented in a box plot using the UCSC Xena. Expression profiles [log2(normalized counts + 1)] of ligands and receptors genes of the PI3K-AKT signaling pathway were further used to perform clustering analysis using Morpheus [28].

### Overall survival analysis

The Kaplan–Meier plotter (http://kmplot.com/analysis) [37] was used to evaluate the effect of the expression of ligands and receptors genes (microarrays data) on patients’ overall survival. This analysis included two cohorts of patients with serous ovarian cancer, TCGA (*n* = 557) and GSE9891 (*n* = 264). The patients were divided into two groups using the autoselect best cutoff, and only JetSet best probes [38] were used for the analysis. These two groups were compared using a Kaplan–Meier survival plot. The hazard ratio with 95% confidence intervals and log-rank test *P* values were determined.

## Results

### Clinical characteristics of the serous ovarian cancer patients

**Fig. 1:**
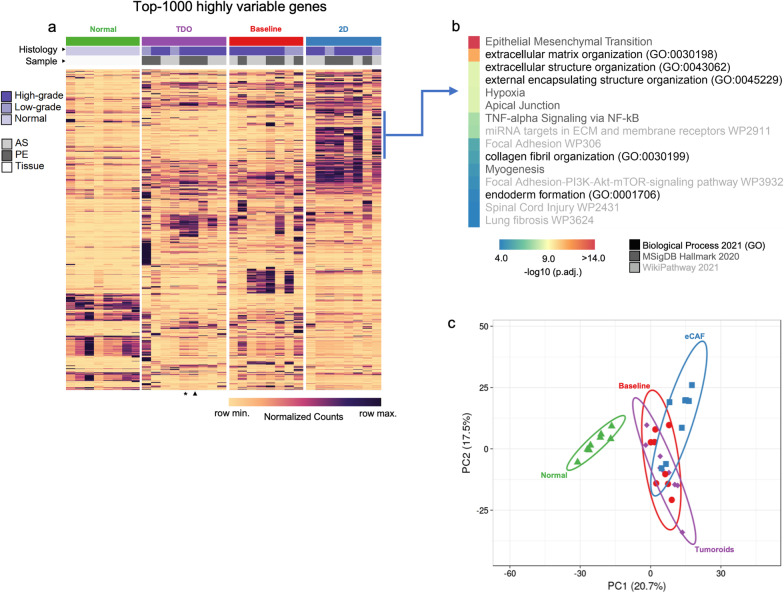
RNA-Sequencing analysis in cells from malignant effusions (baseline), 2D, and tumor-derived organoids (TDO) compared with normal ovarian tissues. **A** Top 1000 highly variable genes (highest standard deviation) across all samples. Rows and columns grouped by each condition (baseline, 2D, TDO, or normal tissue) were clustered based on the Euclidean distance between normalized counts values. In one patient, two TDO were obtained from distinct collections and days in culture (11 and 35 days), and are represented by an arrow and asterisks, respectively, at the bottom of the heatmap. AS: ascites and PE: pleural effusion. **B** Two-dimensional (2D) cell culture system presents a set of genes enriched for epithelial-mesenchymal transition (MSigDB Hallmark 2020), collagen fibril and extracellular matrix (ECM) organization (GO – Biological Process), and Focal Adhesion and PI3K-AKT-mTOR-signaling pathways (WikiPathway 2021). **C** Unsupervised PCA of RNA-Seq data (normalized counts) showing the segregation of ovarian cancer 2D cells and normal tissues from the cluster generated by ovarian cancer-derived organoids and baseline

Eight patients with serous ovarian cancer (FIGO stage III-IV) [39] who underwent drainage of malignant effusions (four pleural effusions and four ascites) with or without neoadjuvant treatment were included in this study (Additional file 14: Table S1). The median age of the patients was 72.5 (37–83) years old. Seven patients deceased of the disease (last follow-up February 2022). Four patients underwent debulking surgery. Six patients received neoadjuvant chemotherapy, and maintenance therapy was applied to four patients. Two patients presented previous colorectal cancer (case 4) and breast cancer (case 5) treated with surgery and surgery and chemotherapy, respectively. These malignant effusion fluids were processed, and cells were seeded in a culture medium to generate 2D cell culture and TDO. In one patient, two TDO were obtained from distinct collections and days in culture (11 and 35 days), which presented similar transcriptional profiles (Fig. 1A). The organoids derived from ovarian tumors presented different growth rates and formed structures with morphology and cohesiveness that varied from dense, low cohesive, or cystic (Additional file 1: Fig. S1), as described by Maenhoudt et al. [40].

Based on the histological analysis of the primary ovarian cancer samples, six patients were diagnosed with high-grade and two with low-grade serous carcinoma (Additional file 14: Table S1). We compared primary tumors and TDO using hematoxylin–eosin staining and immunohistochemical analyzes. The expression of CK7, PAX8, Calretinin, and TP53 in TDO was similar to their corresponding primary tumors (representative example in Additional file 2: Fig. S2).

### RNA-Sequencing reveals that 2D cell culture is enriched for genes involved in the epithelial-mesenchymal transition and extracellular matrix organization

We selected the 1,000 most variable genes among all samples to capture specific biological processes and pathways associated with each condition (Fig. 1A). For most samples, cells from pleural effusions and ascites or from high-grade and low-grade tumors did not cluster closely but were more widely scattered throughout the heatmap (Fig. 1A). We found that the TDO and baseline are clustered in the PCA plot between 2D cells and normal tissues (Fig. 1C). The sequencing data of two TDO obtained from one patient were grouped together with similar expression patterns (Fig. 1A). The 2D cells enriched for a set of genes involved in the epithelial-mesenchymal transition, organization of collagen fibrils and ECM, focal adhesion and PI3K-AKT-mTOR signaling pathways (Fig. 1B, Additional file 14: Table S3). These data suggest that our 2D cells are enriched with ECM component–secreting cells and constitute a cell culture system enriched with CAFs.

### Single-cell transcriptomics reveals the heterogeneity of cancer-associated fibroblasts (CAFs) in ovarian cancer ascites

**Fig. 2:**
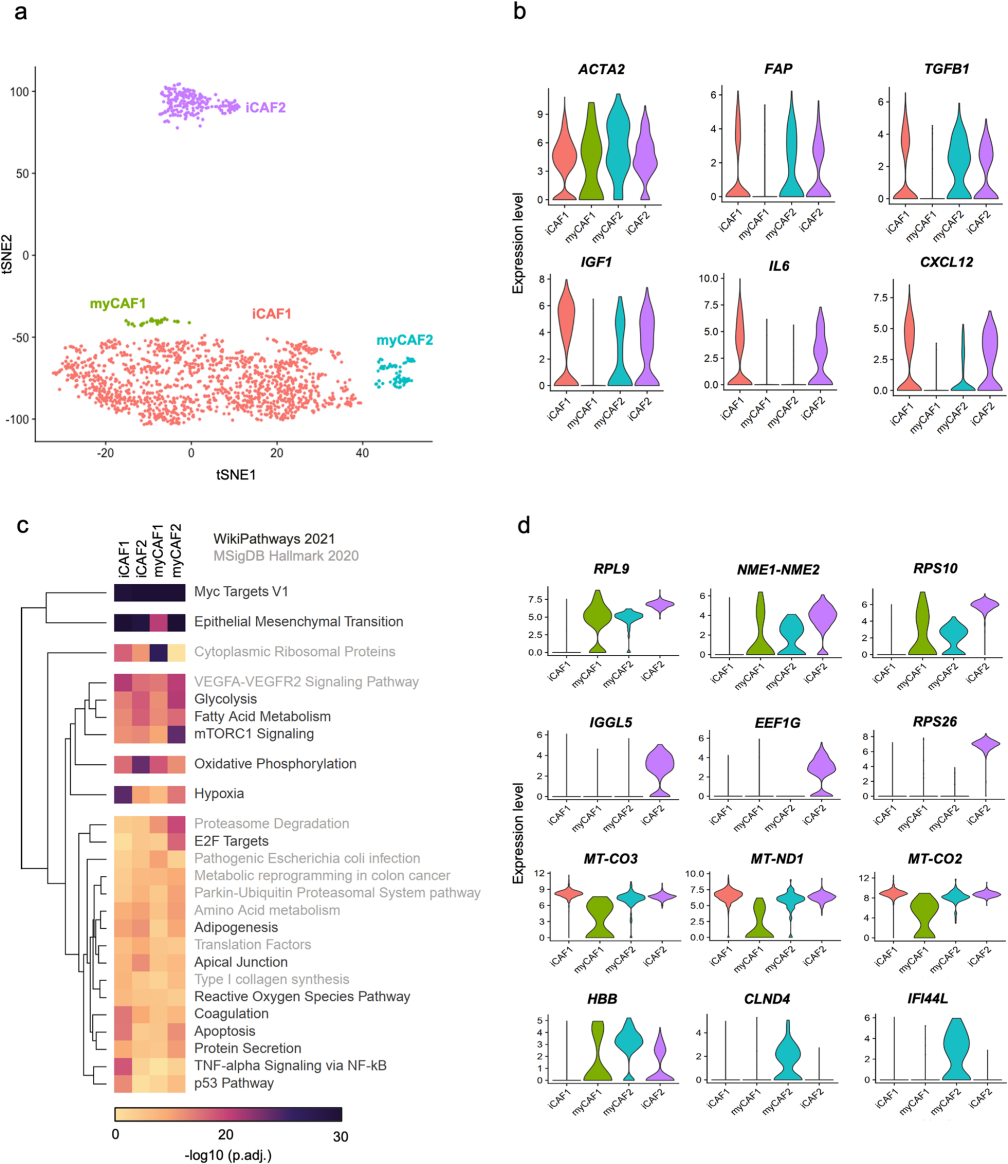
Single-cell transcriptomics re-analysis confirm the heterogeneity of cancer-associated fibroblasts (CAFs) in ovarian cancer malignant ascites. **A** Single-cell RNA-sequencing (scRNA-seq) data visualization of four CAFs populations using t-distributed stochastic neighbor embedding (tSNE). **B** Violin plots show the expression levels of canonical CAFs markers in CAFs populations, including myofibroblastic cancer-associated fibroblasts (myCAF1-2) and inflammatory cancer-associated fibroblasts (iCAF1-2). **C** Heatmap shows the pathway enrichment analysis (EnrichR [30, 31]) for gene sets of cluster-specific CAFs markers. Top 25 pathway terms (lowest adjusted *P*-value) selected from the WikiPathways 2021 and MSigDB Hallmark 2020 libraries available in EnrichR [30, 31]. **D** Violin plots show the expression levels of ovarian cancer CAFs markers among the four CAFs populations

We integrated our bulk RNA-Seq data with the publicly available scRNA-Seq data of malignant ascites from HGSOC patients [17]. First, we followed the single-cell pipeline described by the authors [17] to reanalyze this HGSOC scRNA-Seq dataset and generate the same 18 clusters of cells (Additional file 14: Tables S4 and S5). These clusters include ovarian cancer cells (Ep), myofibroblastic cancer-associated fibroblasts (myCAF), inflammatory cancer-associated fibroblasts (iCAF), macrophages (mac), dendritic cells (DC1), B cells (B), T cells (T), and erythrocytes (ery) (Additional file 3: Fig. S3, Additional file 14: Tables S4 and S5). Next, we confirmed the expression of canonical CAFs markers to define four clusters that include myCAF (myCAF1 and myCAF2) and iCAF (iCAF1 and iCAF2) between all cell types (Fig. 2A and 2B, Additional file 14: Tables S6 and S7). All CAFs clusters expressed *ACTA2,* while *FAP, TGFB1,* and *IGF1* were expressed in iCAF1, myCAF2, and iCAF2 (Fig. 2B). The markers *CXCL12* and *IL6* were highly expressed in iCAF1 and iCAF2 but expressed at low levels in myCAF2 (Fig. 2B). These findings confirmed the enrichment of CAFs in our 2D system and were used to estimate the mechanisms of CAFs communication and interactions with cancer cells.

A gene set enrichment analysis using cell markers was performed to characterize pathways exclusively associated with CAFs subtypes (Fig. 2C). All CAFs were strongly enriched for MYC targets, epithelial-mesenchymal transition pathway, VEGFA-VEGFR2 signaling pathway, glycolysis, fatty acid metabolism, and mTOR1 signaling (Fig. 2C). The iCAF1 cluster featured genes related to hypoxia and TNF-alpha signaling via NF-KB, while the iCAF2 were associated with oxidative phosphorylation (Fig. 2C). myCAF2 showed a specific enrichment of genes related to mTORC1 signaling, proteasome degradation, and E2F targets (Fig. 2C), while myCAF1 presented an enrichment of genes associated with cytoplasmic ribosomal proteins.

The top DEGs capable of distinguishing the four clusters of CAFs were then identified (Fig. 2D, Additional file 14: Table S8). iCAF1 cluster presented reduced expression of *RPL9*, *NME1-NME2,* and *RPS10*, while *IGGL5*, *EEF1G,* and *RPS26* were expressed exclusively in iCAF2 (Fig. 2D). myCAF1 exhibited reduced expression of the mitochondrial genes *MT-CO3*, *MT-ND1,* and *MT-CO2,* while myCAF2 was marked by increased expression of *HBB*, *CLND4,* and *IFI44L* (Fig. 2D)*.*

### Cancer-associated fibroblasts share common outgoing communication patterns for PI3K-AKT signaling pathway and focal adhesion in malignant effusions from ovarian cancer patients

**Fig. 3:**
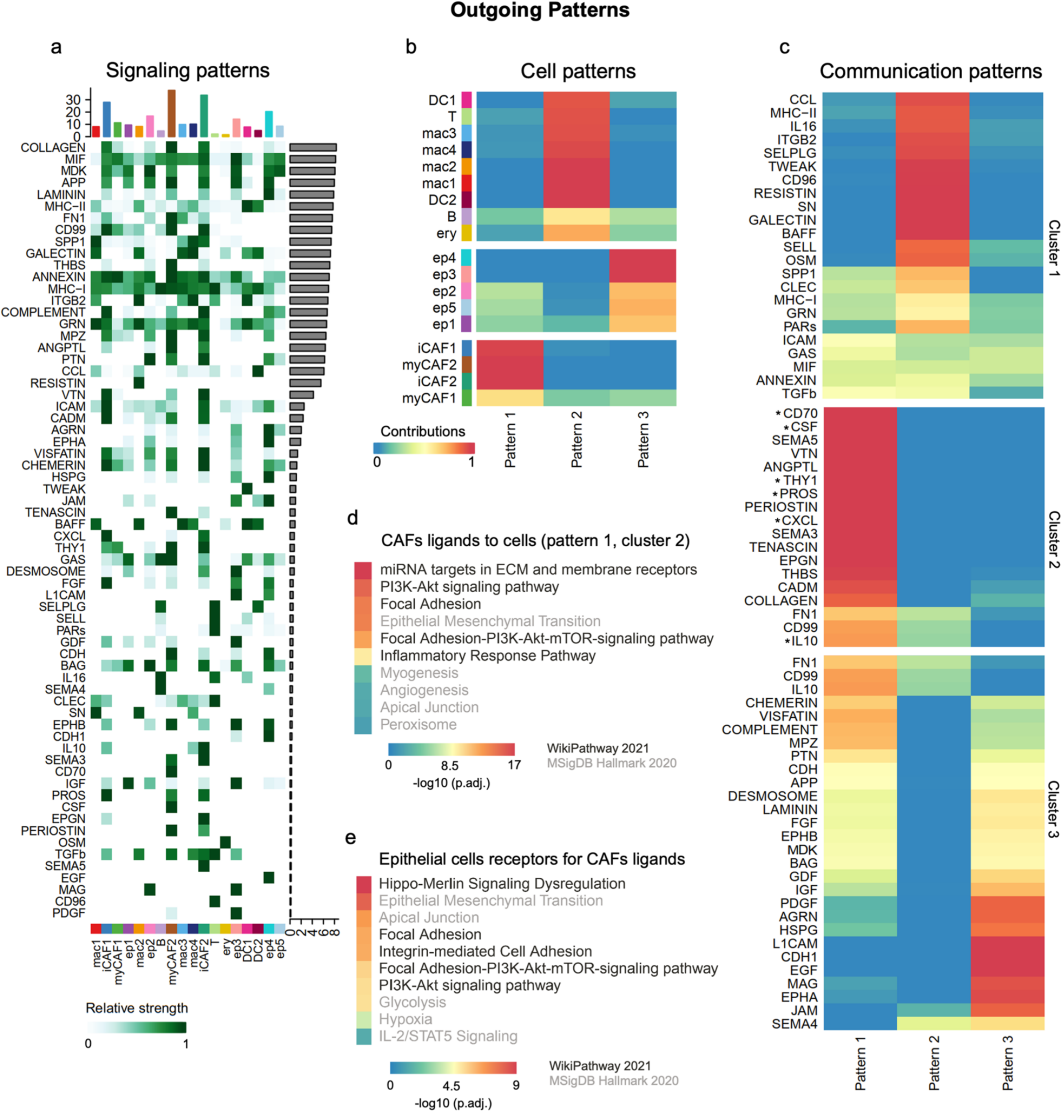
Cancer-associated fibroblasts (CAFs) share common outgoing communication patterns for the PI3K-AKT signaling pathway and focal adhesion in malignant effusions from ovarian cancer patients. Outgoing patterns for signaling **A**, cell **B**, and communication **C** of multiple cell types in malignant abdominal fluids of ovarian cancer patients obtained with CellChat [35] from scRNA-seq data [17]. The asterisks in **C** indicate pathways not associated with CAFs and epithelial cells communication (pattern 1, cluster 2). This communication pattern 1 (cluster 2) was used to identify pathways for gene sets of CAFs ligands **D** and epithelial cell receptors **E** for these ligands. In both **D** and **E**, the focal adhesion and PI3K-AKT-mTOR are among the top altered pathways. The top 10 pathway terms (lowest adjusted *P*-value) were selected from WikiPathways 2021 and MSigDB Hallmark 2020 libraries available in EnrichR [30, 31]

We used CellChat [35] to explore putative cellular interactions and communication (receptor-ligand pairs) between CAFs and other cells in malignant effusion fluids of HGSOC patients evaluated by scRNA-Seq (Additional file 14: Table S9). This analysis included cellular interactions and communication based on secreted signaling (61.8%), extracellular matrix-receptor (21.7%), and cell–cell contact genes (16.5%) (Additional file 4: Fig. S4A). iCAF1, myCAF2, and iCAF2 are cells that contribute the most to outgoing signals (Fig. 3A, Additional file 4: Fig. S4B). Collagens, MIF, MDK, APP, and laminin are significantly involved in cellular interaction and communication for outgoing signals (Fig. 3A).

We used a method of analysis of gene expression patterns available on CellChat to explore how cells and signaling pathways globally coordinate to function. First, we identified the correspondence between inferred latent communication patterns with groups of secreting cells to decipher outgoing communication patterns. Three signaling patterns were found (Additional file 5: Fig. S5A), each of them associated with specific cell types that contribute mainly to the outgoing communication: pattern #1 (CAFs), pattern #2 (immune and blood cells), and pattern #3 (epithelial cancer cells) (Fig. 3B). Then, to identify the pathways associated particularly with outgoing communication shared by the four CAFs subtypes, we selected the list of all CAFs ligands (Additional file 14: Table S9) found in the pathways most contributing to communication pattern 1 (cluster 2; Fig. 3C). These CAFs-specific ligands enriched terms related to miRNA targets in ECM and membrane receptors, PI3K-AKT signaling, focal adhesion, epithelial-mesenchymal transition, and focal adhesion-PI3K-AKT-mTOR-signaling pathway (Fig. 3D). Epithelial cancer cell receptors for these CAFs ligands highlight a similar set of epithelial-mesenchymal transition, PI3K-AKT, and focal adhesion-related pathways as essential mediators of crosstalk between CAFs and cancer cells (Fig. 3E).

### Cancer-associated fibroblasts receive a specific signaling pattern associated with the differentiation pathway and EGFR tyrosine kinase inhibitor resistance

**Fig. 4:**
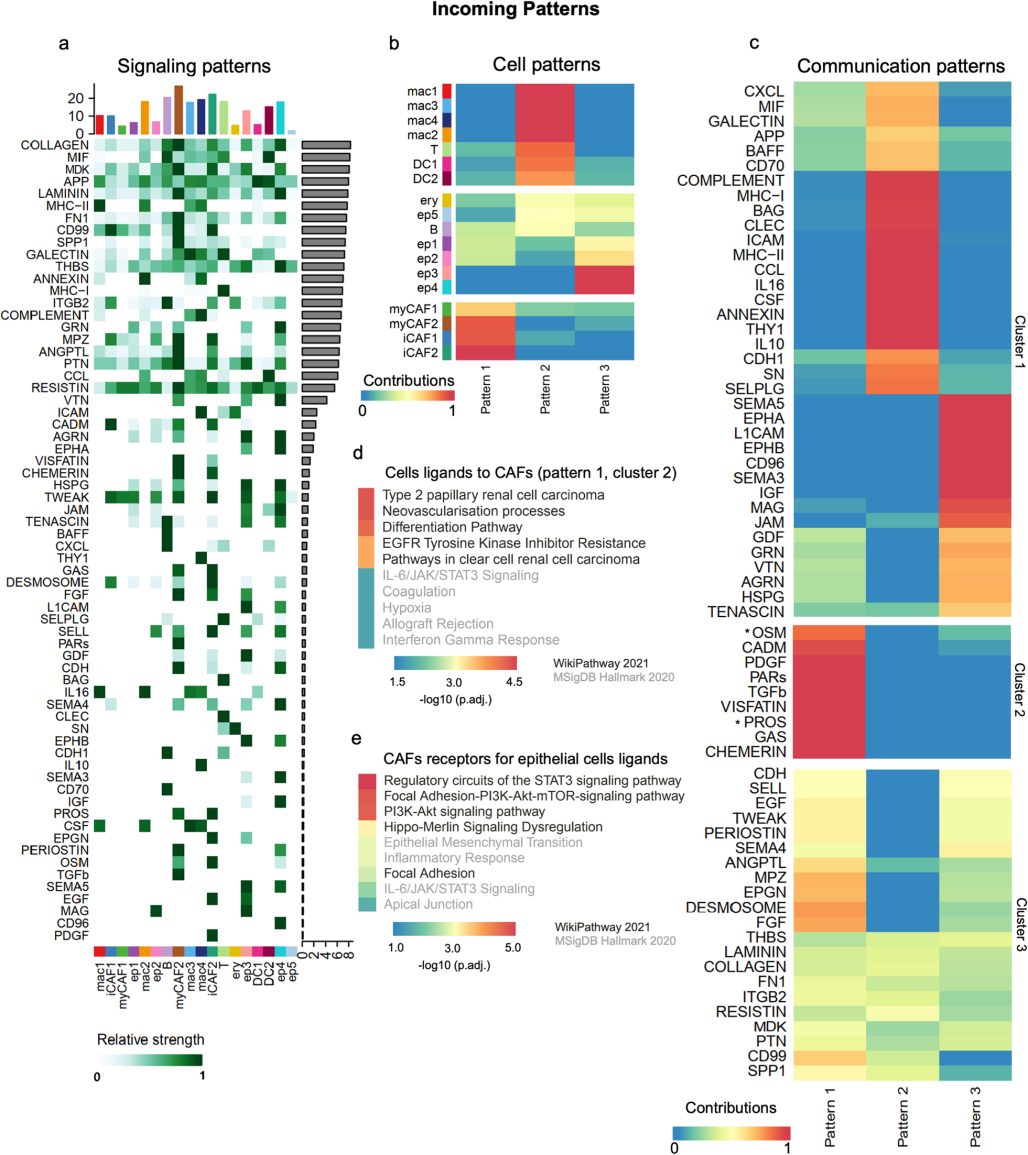
Cancer-associated fibroblasts (CAFs) receive a specific communication pattern from malignant effusion cells associated with the differentiation pathway and EGFR tyrosine kinase inhibitor resistance. Incoming patterns for signaling **A**, cell **B**, and communication **C** of multiple cell types in malignant abdominal fluids of ovarian cancer patients obtained with CellChat [35] from scRNA-seq data [17]. The asterisks in **C** indicate pathways not associated with CAFs and epithelial cells communication (communication pattern 1, cluster 2). This communication pattern 1 (cluster 2) was used to identify pathways for gene sets of cells ligands **D** and CAFs receptors for these ligands **E**. In both **D** and **E**, the focal adhesion-PI3K-AKT-mTOR are among the top altered pathways. The top 10 pathway terms (lowest adjusted *P*-value) were selected from WikiPathways 2021 and MSigDB Hallmark 2020 libraries available in EnrichR [30, 31]

We also identified signals that most contribute to the incoming signaling to cell populations in malignant ascites (CellChat) [35]. Based on the overexpression of ligands and receptors in all cells, we verified that myCAF2 and iCAF2 are among the leading receivers of most incoming signaling, followed by B cells, cancer cells, and macrophages (Fig. 4A). Collagens, MIF, MDK, APP, and laminin were detected as the most significant incoming signaling molecules (Fig. 4A). We found three incoming signaling patterns (Fig. 4B and Additional file 5: Fig. S5B), in which all CAFs are highly associated with signaling pattern #1 (Fig. 4B). The cells ligands to CAFs (Additional file 14: Table S9) found in the communication pattern #1 (cluster 2; Fig. 4C) were enriched for neovascularization processes, differentiation pathway, EGFR tyrosine kinase inhibitor resistance, and IL-6/JAK/STAT3 signaling (cluster 2, Fig. 4D). CAFs receptors for epithelial cancer cell ligands are consistently involved in the PI3K-AKT and focal adhesion-related pathways (Fig. 4E).

### Top interactions between cancer-associated fibroblasts and epithelial cells

**Fig. 5:**
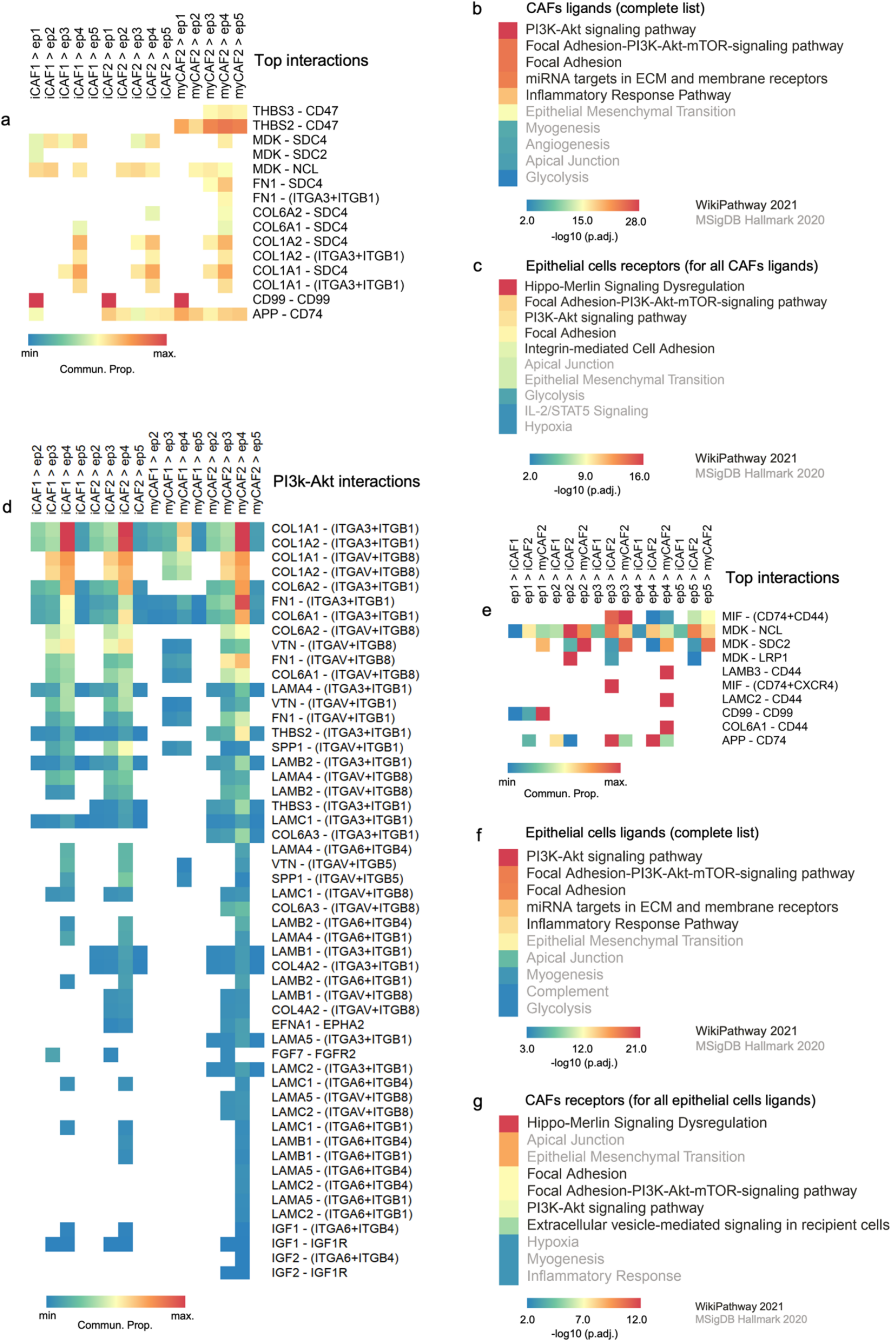
Top interactions between cancer-associated fibroblasts (CAFs) and epithelial cells have multiple ligands and receptors associated with the PI3K-AKTsignaling pathway and focal adhesion. **A** Top ligand-receptor interactions (Commun. Probability > 0.10) between CAFs and epithelial cells. Heatmaps show the EnrichR pathway enrichment analysis for gene sets that define all CAFs ligands **B** and epithelial cell receptors **C**. The top 10 pathway terms (lowest adjusted *P*-value) were chosen from WikiPathways 2021 and MSigDB Hallmark 2020 libraries available in EnrichR [30, 31]. **D** PI3K-AKT ligand-receptor interactions between CAFs and epithelial cells. **E** Top ligand-receptors interactions (Commun. Probability > 0.10) between epithelial cells and CAFs. Heatmaps showing the EnrichR pathway enrichment analysis for gene sets that define all epithelial cells ligands **F** and CAFs receptors **G**. The top 10 pathway terms (lowest adjusted *P*-value) were chosen from WikiPathways 2021 and MSigDB Hallmark 2020 libraries available in EnrichR [30, 31]. Commun. Prob., communication probability

The high number of ligand and receptor interactions (Additional file 6 Additional files: 7,8 and 9: Fig. S6A-D) indicated the complexity of the process that extended to and potentially beyond pattern #1 for outgoing and incoming signaling (cluster 2; Fig. 3C to Fig. 4C). Thus, to explore the top interactions involved in ovarian cancer, we first selected the outgoing signaling from CAFs to epithelial cancer cells with a communication probability > 0.10. Among these, we found interactions that may constitute potential targets for CAFs-based therapies, such as THBS2/THBS3 (myCAF2) and CD47 (cancer cells) or between MDK (CAFs) and SDC2/SDC4/NCL (cancer cells) (Fig. 5A).

A comprehensive evaluation of all ligands and receptors interactions between CAFs and epithelial cells (Additional file 6 Additional files: 7, 8 and 9: Fig. S6A-D) confirmed the crucial role of components of the focal adhesion-PI3K-AKT-mTOR signaling pathway (Fig. 5B–C). These interactions between CAFs and cancer cells (Additional file 6 Additional files: 7,8 and 9: Fig. S6A-D) were filtered, and a high-resolution map with the most relevant interactions of the PI3K-AKT pathway revealed the involvement of collagens, fibronectin, vitronectin, laminin, and osteopontin from CAFs with integrins in cancer cells (Fig. 5D).

CAFs also have an essential role in maintaining an inflammatory tumor environment by secreting cytokines, chemokines, and ECM proteins that recruit and activate different immune effector cells [41]. We used CellChat to infer and analyze CAFs-immune cells communication using single-cell data (Additional file 14: Table S9). The bi-directional crosstalk between CAFs and immune cells further confirmed that the PI3K-AKT signaling pathway is a general marker of the CAFs signaling (CAFs ligands and receptors) with other cell types (Additional file 10: Fig. S7).

### MIF and MDK are essential regulators of cancer-associated fibroblasts (CAFs) by epithelial cancer cells

We speculate that the diffusion of ligands produced by epithelial cancer cells into the adjacent stroma may also stimulate CAFs. The top outgoing signaling from epithelial cancer cells to CAFs (communication probability > 0.10) showed interactions between MIF (cancer cells) and CD74 + CD44 (CAFs) or MDK (cancer cells) and NCL/SDC2/LRP1 (CAFs) (Fig. 5E). In concordance with previous results herein described, both epithelial cancer cell ligands and CAFs receptors were enriched for PI3K-AKT and focal adhesion-related pathways. These ligands were highly correlated with their receptors (and vice versa) with redundancies for these top-enriched pathways (Fig. 5F–G).

### Relevance of ligands and receptors genes associated with the PI3K-AKT signaling pathway in ovarian cancer

**Fig. 6:**
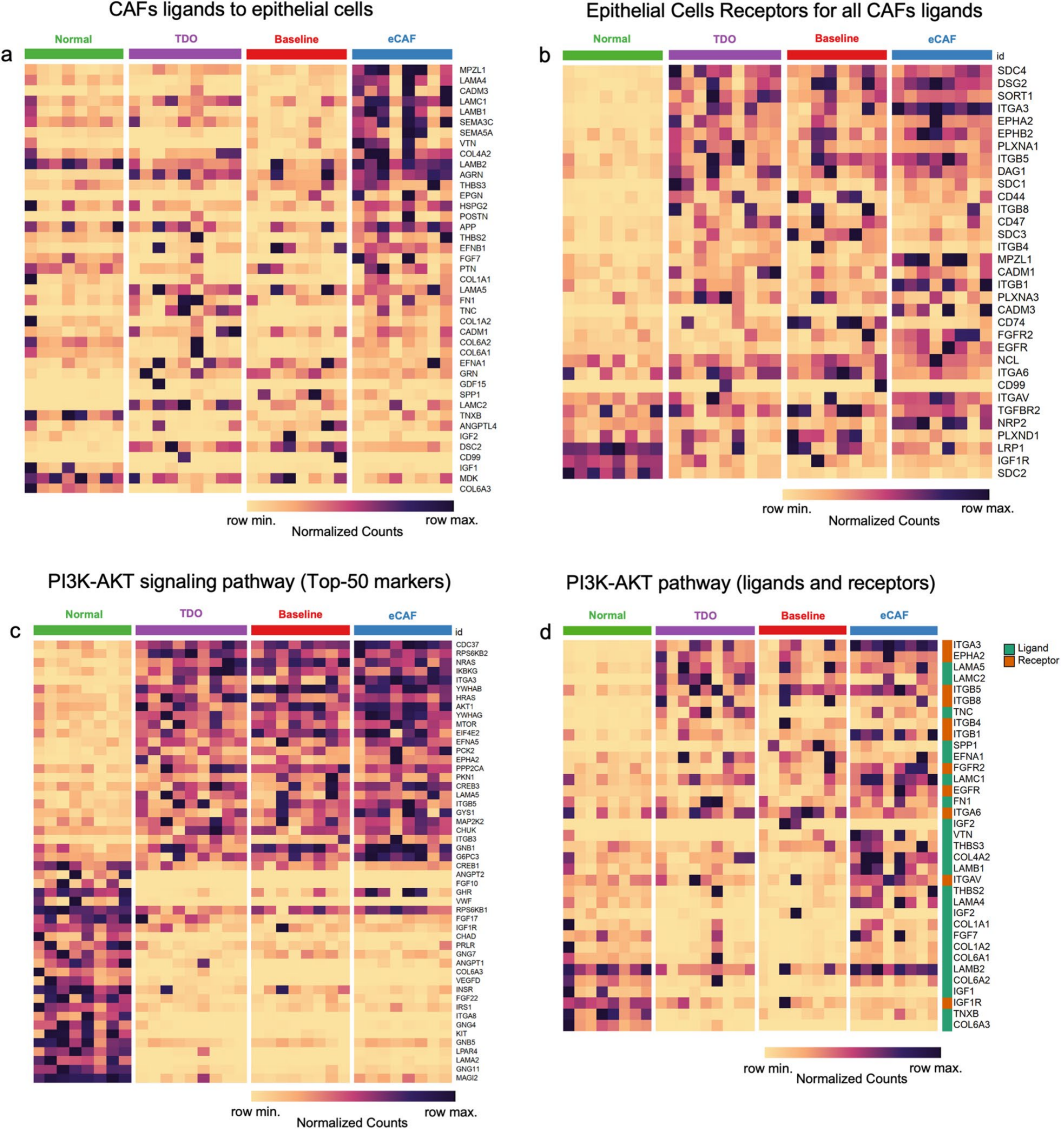
Establishment of in vitro system models based on tumor-derived organoids and culture enriched with cancer-associated fibroblasts (eCAFs) to validate the expression profile of ligand and receptor genes associated with the PI3K-AKT signaling pathway in ovarian cancer. Gene expression of CAFs ligands **A** and epithelial cells receptors **(B)** for these ligands. Genes were ordered based on a marker selection (signal to noise) that highlights the differences in the expression profile (normalized counts) of eCAFs **A** or normal tissues **B** compared to the other three conditions. **C** Top 50 highly variable genes from the PI3K-AKT signaling pathway across all samples. Genes were ordered based on a marker selection (signal to noise) that highlights the differences in the expression profile (normalized counts) between normal tissue and malignant cells. **D** Expression profile (normalized counts) of ligands and receptors of the PI3K-AKT signaling pathway across all samples. Rows (for ligand or receptor genes) were ordered based on a marker selection (signal to noise) that highlights the differences in the expression profile (normalized counts) of normal tissues when compared to the other three conditions

We explored the PI3K-AKT signaling pathway using our RNA-Seq data performed in malignant fluid cells from eight HGSOC patients. This analysis included the baseline, 2D cells, and TDO combined with normal ovarian tissues (Additional file 14: Table S10). First, we confirmed that our 2D cells were eCAFs and captured the similar expression profiles found in the scRNA-Seq re-analysis. To this, we analyzed the expression profile of the top ten markers that distinguished each CAFs cluster from the other ascites cells of the scRNA-Seq re-analysis (Additional file 11: Fig. S8). The expression profile of these marker genes did not show differences between pleural effusion and ascites, nor between high- and low-grade samples (Additional file 11: Fig. S8). Next, we analyzed the expression profile of CAFs ligands targeting epithelial cells (Fig. 6A) or CAFs ligands within pattern #1 (from cluster 2, in Fig. 3C) (Additional file 12: Fig. S9), and both showed a clear enrichment of CAFs outgoing signaling. However, compared to normal tissues, epithelial cell receptors were enriched in eCAFs, TDO, and baseline (Fig. 6B). The profile of cell receptors indicated a complex regulatory interplay based on CAFs paracrine and autocrine signaling (Fig. 6B, Additional file 4: Fig. S4B). CAFs seem to act through ligands that stimulate an increase in the expression of the PI3K-AKT genes in epithelial cancer cells, including downstream effector genes such as *AKT1* and *MTOR* (Fig. 6C). These downstream PI3K-AKT effector genes were consistently increased in baseline, TDO, and eCAF (Fig. 6C). Also, many of the PI3K-AKT ligand genes showed increased expression explicitly in eCAFs (Fig. 6D).

### Increased expression of genes involved in PI3K-AKT signaling between cancer-associated fibroblasts and cancer epithelial cells is associated with worse overall survival in ovarian cancer patients

The expression profile of the ligands and receptor genes of the PI3K-AKT signaling pathway in the CAFs-cancer cell crosstalk revealed genes previously associated with the outcome, response to treatment, or overall survival in ovarian cancer (Fig. 6d). This list of 34 genes includes components of *ITGA* and *ITGB* superfamily [42], laminins [43], fibroblast growth factors [44, 45], collagens [22, 46], insulin growth factors [47, 48], *EGFR* [49, 50], *FN1* [22, 51–53], *EPHA2* [54], *TNC* [55], SPP1 [55, 56], and VTN [22, 57].

**Fig. 7:**
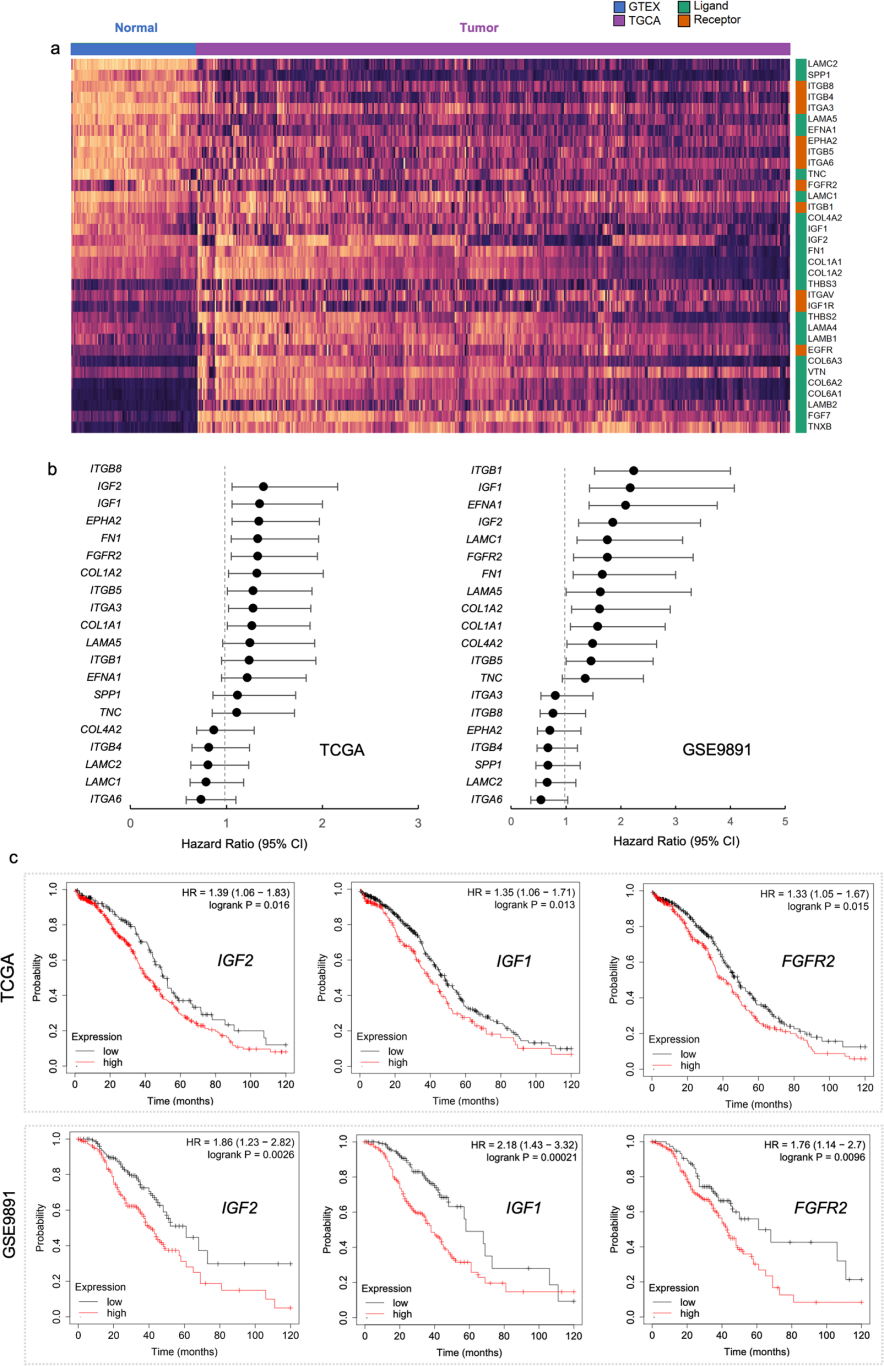
Increased expression of genes involved in PI3K-AKT signaling in cancer-associated fibroblasts and cancer epithelial cells from ovarian cancer patients is associated with worse overall survival. **A** Expression levels [log2(norm_count + 1)] of ligands and receptors of the PI3K-AKT signaling pathway in ovarian cystadenocarcinoma (*n* = 419) of The Cancer Genome Atlas (TCGA) compared to normal ovarian tissues (*n* = 88) tissues of Genotype-Tissue Expression (GTEx). Rows (for ligand or receptor genes) were ordered based on a marker selection (signal to noise) that highlights the differences in the expression profile [log2(norm_count + 1)] of GTEx tissues when compared to the TCGA tissues. Twenty genes exhibited increased expression levels and were selected to test their association with overall survival. **B** Forest plots for these twenty ligand and receptor genes of the PI3K-AKT signaling pathway in two cohorts of patients with ovarian cancer (TCGA, *n* = 557 and GSE9891, *n* = 264) available on the Kaplan–Meier (KM) plotter [37]. The hazard ratio (HR) with 95% confidence intervals (CI) determined by Cox proportional hazards model was used to evaluate the association between gene expression values and overall survival. The statistical significance was determined by a log-rank test. **C** Representative Kaplan–Meier curves of overall survival for patients with ovarian cancer (TCGA and GSE9891) based on the expression of *IGF2*, *COL1A2*, *IGF1*, and *FGFR2*

We also evaluated the expression levels of these PI3K-AKT pathway ligands and receptors genes by comparing TCGA ovarian cancer cohort (*n* = 418) with GTEx normal samples (*n* = 88). Twenty genes exhibited increased and 14 decreased expression levels in ovarian cancer (Fig. 7A and Additional file 13: Fig. S10). We focused on the 20 up-regulated genes of the PI3K-AKT pathway to test their association with overall survival in two cohorts of patients (TCGA, *n* = 557 and GSE9891, *n* = 264) available on the Kaplan–Meier (KM) plotter [37]. Nine genes from the TCGA dataset and 12 genes from the GSE9891 dataset were significantly associated with a worse overall survival rate (HR > 1 and log-rank *P* value < 0.05) (Fig. 7b-c). Seven of these genes, *COL1A1, ITGB5, COL1A2, FGFR2, FN1, IGF1,* and *IGF2,* predicted worse overall survival in both datasets (Fig. 7B–C).

## Discussion

Increasing evidence indicates that communication and interactions between cancer cells and CAFs are essential determinants of tumor metastasis and progression [12, 13]. The molecular mechanisms involved in these interactions may contribute to the identification of new and more effective therapies or unveil clinically useful prognostic biomarkers for patients with ovarian cancer. We reanalyzed scRNA-seq data from malignant ascites of HGSOC patients [17] to infer and investigate cell–cell interactions and communications. Next, we integrated these gene lists with our RNA-Seq data performed in patient-derived 2D cell culture and tumor organoids. Interestingly, our 2D cells system was enriched with CAFs (eCAFs), as demonstrated by a specific transcriptional signature comprising stromal-related pathways. Our gene expression-based strategy revealed several ligand and receptor interactions between CAFs and cancer epithelial cells associated with the PI3K-AKT signaling pathway and focal adhesion. From the total of 34 ligand and receptor genes identified in these interactions, seven (*COL1A1, ITGB5, COL1A2, FGFR2, FN1, IGF1,* and *IGF2*) were consistently up-regulated and predicted worse overall survival in two additional cohorts (TCGA and GSE9891) of ovarian cancer patients.

CAFs exhibit considerable plasticity and heterogeneity [58], and different subpopulations have been reported in ovarian cancer ascites and tumor tissues. Givel et al. [59] described the stromal heterogeneity in HGSOC with four CAFs subpopulations (CAF-S1 − S4). According to the authors, the accumulation of the CAF-S1 subset was associated with an immunosuppressive tumor environment. On the other hand, Kan et al. [21] identified two subpopulations of CAFs (CAF1 and CAF2), where CAF1 was strongly associated with metastasis. Hussain et al. [60] described two CAFs states (FAP-low and FAP-high) and showed that CAFs FAP-high promote proliferation, invasion, and therapy resistance of cancer cells. Izar et al. [17] identified four CAFs sub-populations, with two expressing immune-related genes (complement factors, chemokines, and cytokines). We demonstrated that between these CAFs expressing immune-related genes (iCAF1 and iCAF2), iCAF1 featured genes related to hypoxia and TNF-alpha signaling via NF-KB, while iCAF2 were highly associated with oxidative phosphorylation (Fig. 2C). We also found that myCAF2 showed a specific enrichment of genes related to mTORC1 signaling and proteasome degradation, and E2F targets, while myCAF1 exhibited reduced expression of mitochondrial genes (Fig. 2C). Importantly, these results showed that CAFs present distinct functional states but also demonstrated that these cells share signaling patterns that can be explored for targeted therapies.

Several studies have shown that the activation of CAFs by cancer cells is mediated via secretome components associated with ECM remodeling, cytokine, chemokines, and growth factor-mediated signaling [58, 61–64]. We found that CAFs from malignant fluids of ovarian cancer receive a specific signaling pattern from cancer cells, mainly composed of ligands associated with neovascularization processes, differentiation pathway, EGFR tyrosine kinase inhibitor resistance, and IL-6/JAK/STAT3 signaling (Fig. 4D). Furthermore, we verified that MIF and MDK are essential regulators of CAFs by epithelial cancer cells targeting the heteromeric complex CD74 + CD44 and NCL/SDC2/LRP1, respectively (Fig. 5E). MIF is known to be produced by ovarian cancer cells in an autocrine manner and may promote colonization of the peritoneum and neovascularization of tumor deposits by other cytokines, chemokines, and growth factors [65]. Furthermore, MIF is secreted in ascites, and its serum levels in patients with ovarian cancer correlate with a poor prognosis [66]. Several therapeutic strategies under development use antibodies to block MIF or CD74 and thus prevent MIF signaling in different tumor types [67]. Therefore, our findings may be valuable not only for helping to explain the role of MIF in ovarian cancer but also for designing targeted therapies that act on specific molecular targets of cancer cells that may activate CAFs.

The release of factors by activated CAFs enormously impacts cancer cells in the crosstalk that occurs in ovarian cancer and is associated with several clinicopathological characteristics and disease outcomes [13, 58]. Among the top CellChat interactions, we identified MDK from CAFs targeting SDC2/SDC4/NCL of cancer cells (Fig. 5A). Thus, MDK may participate in two-way communication between CAFs and ovarian cancer cells and act in an autocrine or paracrine manner. MDK is a growth factor that acts on cancer progression and constitutes a potential therapeutic target [68]. In addition, among these top interactions, we found THBS2/THBS3 secreted by myCAF2 targeting CD47 of cancer cells (Fig. 5A). CD47 is a highly and ubiquitously expressed cell surface protein in ovarian cancer [69] that induces cancer cell growth and predicts poor prognosis [70, 71]. CD47 inhibits macrophage phagocytosis, which contributes to ovarian cancer progression [72, 73]. Consequently, CD47 has been tested in ovarian cancer as a promising CAR-T cell-based therapy [74]. A treatment that combines engineered CAR-T cells targeting CD47 and inhibits secreted THBS2/THBS3 using antibodies may constitute a valuable therapeutic strategy to inhibit ovarian cancer progression.

We show that the interplay between CAFs and epithelial cancer cells has multiple ligand and receptor interactions associated with the PI3K-AKT signaling pathway and focal adhesion (Fig. 5B–C). The activation of this pathway promotes cellular proliferation, migration, and invasion in the ovarian cancer [75]. Our RNA-seq data confirmed the relevance of these ligands and receptors of the PI3K-AKT signaling pathway (Fig. 6). CAFs induce cancer cell proliferation and metastasis by activating the PI3K-AKT-mTOR pathway in lung [76–78], colon [79], gastric [80], oral [81], endometrial [82], and anal [83] cancers. The CAF-derived ligand POSTN (Fig. 6A) found in our study was previously associated with chemoresistance in ovarian cancer [50]. Furthermore, the secretion of FNI by mesothelial cell-derived CAFs was also shown to decrease the platinum sensitivity of ovarian cancer cells by inducing the PI3K-AKT pathway [84]. Drugs targeting the PI3K-AKT signaling pathway, such as BEZ235, AZD5363, and NSC777213, have been tested to treat ovarian cancer with promising results [85–87]. A phase I trial with PI3-kinase inhibitor BKM120 in combination with PARP inhibitor olaparib is being conducted in patients with high-grade serous ovarian cancer, with evidence of clinical benefits [88]. The pharmacological and genetic inhibition of TTK, which is involved in the PI3K-AKT pathway, decreases the proliferation of ovarian cancer cells and increases their sensitivity to cisplatin by suppressing autophagy [89]. Thus, drugs targeting the crosstalk between CAFs and cancer cells by blocking PI3K-AKT ligands and receptors constitute relevant therapeutic targets.

Considering the intercommunication between CAFs and cancer cells based on components of the PI3K-AKT pathway and its impact on mechanisms that induce tumor aggressiveness or drug resistance, we evaluated whether changes in expression of these ligands and receptors genes are associated with prognosis. Several genes found in our analysis have previously been described as biomarkers of ovarian cancer or potential therapeutic targets, including genes from the *ITGA* and *ITGB* superfamily [42], laminins [43], fibroblast growth factors [44, 45, 90], collagens [22, 46], insulin growth factors [47, 48], *EGFR* [49, 50], *FN1* [22, 51–53], *EPHA2* [54], *TNC* [55], *SPP1* [55, 56], and *VTN* [22, 57]. We demonstrated that *COL1A1, COL1A2, FGFR2, FN1, IGF1, IGF2,* and *ITGB5* were also altered in two large cohorts of patients (TCGA and GSE9891) and are highly relevant for predicting the prognosis (Fig. 7B). These data demonstrate that CAFs induce changes in cancer cells via components of the PI3K-AKT pathway associated with ECM and adhesion. These results are aligned with findings showing that matrix adhesion is an adaptative response that drives HGSC aggressiveness by co-evolving ECM composition and sensing [22].

*COL1A1* plays a central role in carboplatin resistance in ovarian cancer, acting through the ECM-receptor interaction and focal adhesion pathways [46]. In addition to contributing to changes in the PI3K-AKT pathway in ovarian cancer, these interactions may promote peritoneal metastasis by forming ascitic CAFs heterotypic aggregates with tumor cells [91]. Integrins such as *ITGB1, ITGB3, ITGB6, ITGA7*, and *ITGB8* are also involved in cell adhesion and signaling and provide further prognostic information and druggability in HGSOC [42]. We found that the integrin *ITGA3* predicted a worse prognosis in the TCGA cohort, *ITGB1* in the *GSE9891* cohort*,* while *ITGB5* predicted a worse prognosis in both. Despite differences in study design and population, we and Zhu et al. [42] highlighted the prognostic value of ITGA and ITGB superfamily members in serous ovarian cancer.

RNA-Seq data provide unprecedented opportunities to explore new biological questions. Although our strategy has limitations, including the number of cases investigated, the ligand-receptor interactions were validated in two independent ovarian cancer cohorts and in a large number of cell types, strengthening the findings described here. In addition to identifying ovarian cancer biomarkers, we elaborated a detailed map of ligand-receptor interactions in ovarian cancer malignant fluids.

In conclusion, we characterized the interactions and communication between CAFs and cancer cells from ovarian cancer ascites using scRNA-Seq data. This analysis was used to compare and generate ligands and receptors from RNA-Seq data performed in patient-derived eCAFs and tumor organoids obtained from ovarian cancer malignant effusions. PI3K-AKT signaling mediating through ligands and receptors potentially constitutes major players in CAFs interactions and communication with cancer cells. We also verified that a set of ligands and receptor genes of the PI3K-AKT pathway presented potential prognostic value. The expression profile of these drivers at the single-cell level revealed molecular mechanisms and targets that may facilitate the development of therapies focusing on interactions and communication between CAFs and cancer cells.

## Supplementary Information

**Additional file 1: Fig. S1. **Tumor-derived organoids (TDO) from malignant effusions of serous ovarian cancer patients. Representative examples of TDO from cases 8 (**A**), 4 (**B**), and 1 (**C**) show differences in the growth rate and morphology. (**D**) Representative images of TDO morphology (dense, low cohesive, and cystic, as described by Maenhoudt et al. [40]) of individual TDO from different patients (cases 4, 7, and 6, respectively) (40x).

**Additional file 2: Fig. S2. **(**A**) High-grade serous carcinoma showing strong and diffuse immunohistochemical expression of CK7, TP53, and PAX8. (**B**) Section from tumor-derived organoid day 11. The atypical cells show strong and diffuse immunohistochemical expression of CK7, TP53, and PAX, similarly to the primary tumor shown in (a). (**C**) Low-grade serous ovarian cancer shows a strong and diffuse immunohistochemical expression of CK7 and PAX8 and absent reaction for calretinin. (**D**) Tumor-derived organoids show atypical cells with strong and diffuse immunohistochemical expression of CK7 and PAX and an absence of reaction for calretinin, similarly to the primary low-grade serous ovarian cancer shown in (**C**).

**Additional file 3: Fig. S3. **Re-analysis based on single-cell RNA-seq data from malignant ascites of eight patients with advanced high-grade serous ovarian cancer described by Izar et al. [17]. The t-SNE shows 9.609 cells analyzed in 18 clusters (colors) that include ovarian cancer cells (Ep1-5), myofibroblastic cancer-associated fibroblasts (myCAF1-2), inflammatory cancer-associated fibroblasts (iCAF1-2), macrophages (mac1-4), dendritic cells (DC1-2), B cells (**B**), T cells (T), and erythrocytes (ery).

**Additional file 4: Fig. S4. (A) **Literature-supported ligand-receptor interactions in humans available at the CellChatDB database (http://www.cellchat.org/) [35]. The 1,939 validated interactions included paracrine/autocrine signaling (61.8%), extracellular matrix (ECM) receptor interactions (21.7%) and cell-cell contact interactions (16.5%). (**B**) Circos plot showing the CellChatDB inferred cell-cell communication network based on single-cell RNA-Seq data across ovarian cancer cells (Ep1-5), myofibroblastic cancer-associated fibroblasts (myCAF1-2), inflammatory cancer-associated fibroblasts (iCAF1-2), macrophages (mac1-4), dendritic cells (DC1-2), B cells (**B**), T cells (T), and erythrocytes (ery) from ovarian cancer patients.

**Additional file 5: Fig. S5. **Cophenetic and Silhouette metrics were used in CellChatDB [35] to identify the number of outgoing (**A**) and incoming (**B**) communication patterns that cell groups and pathways coordinate to function. Both Cophenetic and Silhouette values suggest three patterns, as indicated by the first sudden drop of the measured score in this number of patterns.

**Additional file 6: Fig. S6. **Ligand-receptor interactions between cancer-associated fibroblasts and epithelial cells inferred by CellChatDB [35] using single-cell RNA-seq data [17] from malignant ascites of ovarian cancer patients. (**A**) Inflammatory cancer-associated fibroblasts 1 (iCAF1)-epithelial cells.

**Additional file 7: Fig. S6. (B) **Inflammatory cancer-associated fibroblasts 2 (iCAF2)-epithelial cells.

**Additional file 8: Fig. S6. (C)** Myofibroblastic cancer-associated fibroblasts 1 (myCAF1)-epithelial cells.

**Additional file 9: Fig. S6. (D) ** Myofibrobastic cancer-associated fibroblast 2 (myCAF2)-epithelial cells. 

**Additional file 10: Fig. S7. **Pathways associated with the signaling (ligands and receptors) from CAFs to immune cells (a and b) and from immune cells to CAFs (d and e). The top 10 pathway terms (lowest adjusted *P*-value) were selected from WikiPathways 2021 and MSigDB Hallmark 2020 libraries available in EnrichR [30, 31].

**Additional file 11: Fig. S8. **Expression profile of 40 cancer-associated fibroblast markers (top 10 for each cluster from scRNA-Seq data) in ovarian bulk RNA-Seq. Genes were ordered based on a marker selection (signal to noise) that highlights the differences in the expression profile (normalized counts) between ovarian cancer-associated fibroblasts-enriched culture (eCAFs) and the other three conditions (Normal tissue, Normal; tumor-derived organoids, TDO; and baseline). (**A**) grade; (**B**) malignant effusions or normal tissue; and (**C**) experimental conditions.

**Additional file 12: Fig. S9. **Expression profiling of CAFs ligands (communication pattern 1) in ovarian bulk RNA-Seq. Genes were ordered based on a marker selection (signal to noise) that highlights the differences in the expression profile (normalized counts) between ovarian cancer-associated fibroblasts-enriched culture (eCAFs) and the other three conditions (Normal tissue, Normal; tumor-derived organoids, TDO; and baseline).

**Additional file 13: Fig. S10. **Box plot showing the expression levels [log2(norm_count+1)] of ligands and receptors of the PI3K-AKT signaling pathway in ovarian cystadenocarcinoma (*n* = 419) of The Cancer Genome Atlas (TCGA) compared to normal ovarian tissues (*n* = 88) tissues of Genotype-Tissue Expression (GTEx).

**Additional file 14:** Supplementary Tables (S1-S10).

## Data Availability

The dataset generated during the current study is available from the corresponding author upon reasonable request. Previously published droplet-based scRNA-Seq data for HGSOC ascites samples have the accession number GSE146026.

## Abbreviations

CAFs: Cancer-associated fibroblasts
eCAFs: Patient-derived cancer-associated fibroblasts-enriched
HGSOC: High-grade serous ovarian cancer
ECM: Extracellular matrix
scRNA-Seq: Single-cell RNA sequencing
EMT: Epithelial-mesenchymal transition
RNA-Seq: RNA-sequencing
TCGA: The Cancer Genome Atlas
GTEx: Genotype-Tissue Expression
2D: Two-dimensional
3D: Three-dimensional
TDO: Tumor-derived organoids
H&E: Hematoxylin and eosin
tSNE: T-distributed stochastic neighbor embedding
DEGs: Differentially expressed genes
Ep: Ovarian cancer cells
myCAF: Myofibroblastic cancer-associated fibroblasts
iCAF: Inflammatory cancer-associated fibroblasts
mac: Macrophages
DC1: Dendritic cells
B: B cells
T: T cells
ery: Erythrocytes
KM: Kaplan–Meier
GEO: Gene Expression Omnibus
