# Supplementary figures and images for: Single-cell and bulk RNA sequencing reveal ligands and receptors associated with worse overall survival in serous ovarian cancer
Source: Cell Commun Signal. 2022 Nov 9;20:176. doi: 10.1186/s12964-022-00991-4 (PMC9648056; doi:10.1186/s12964-022-00991-4)

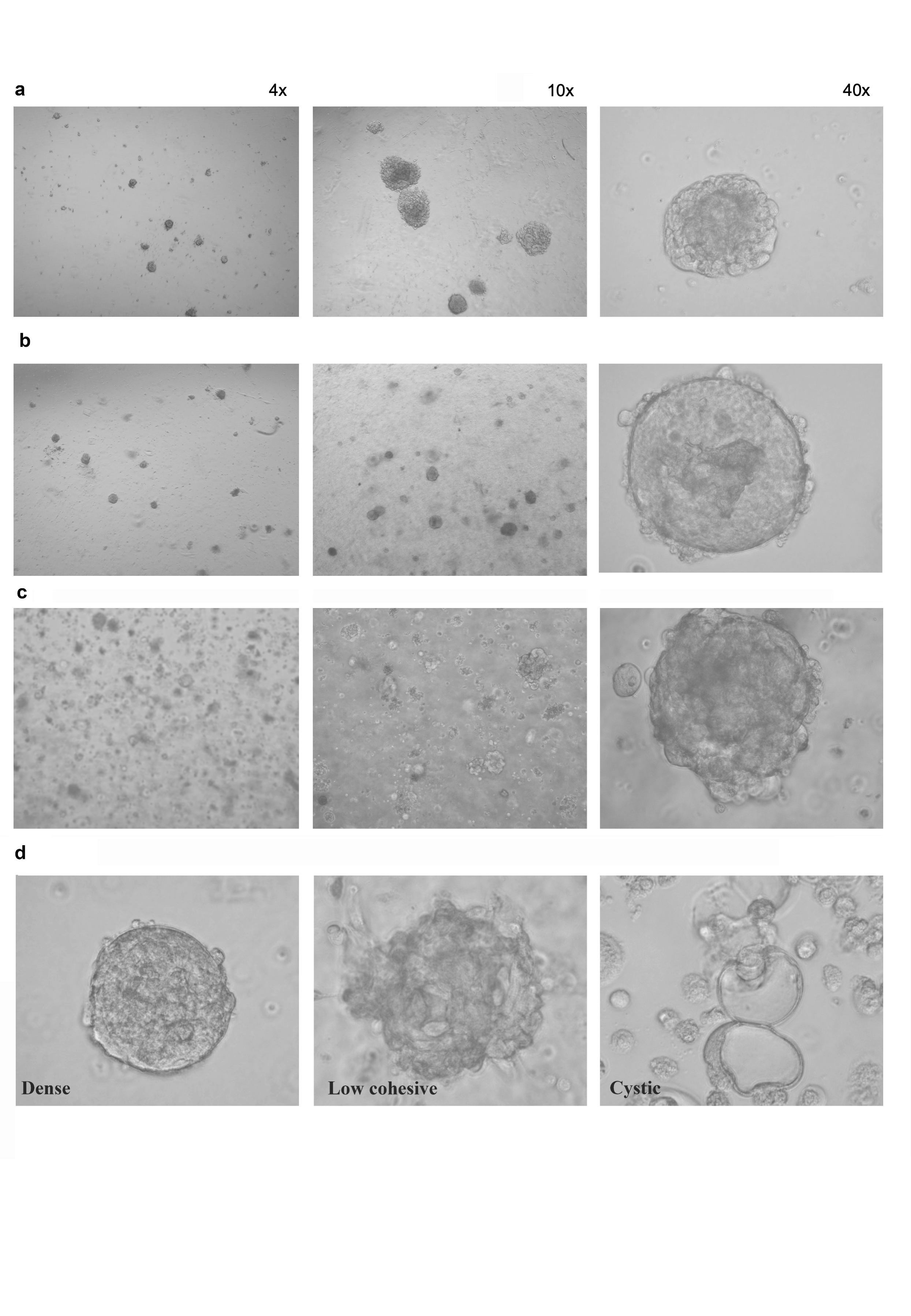

Supplement: Supplementary file 2 — Additional file 1: Fig. S1. Tumor-derived organoids (TDO) from malignant effusions of serous ovarian cancer patients. Representative examples of TDO from cases 8 (A), 4 (B), and 1 (C) show differences in the growth rate and morphology. (D) Representative images of TDO morphology (dense, low cohesive, and cystic, as described by Maenhoudt et al. [40]) of individual TDO from different patients (cases 4, 7, and 6, respectively) (40x). [file 12964_2022_991_MOESM2_ESM.tiff]

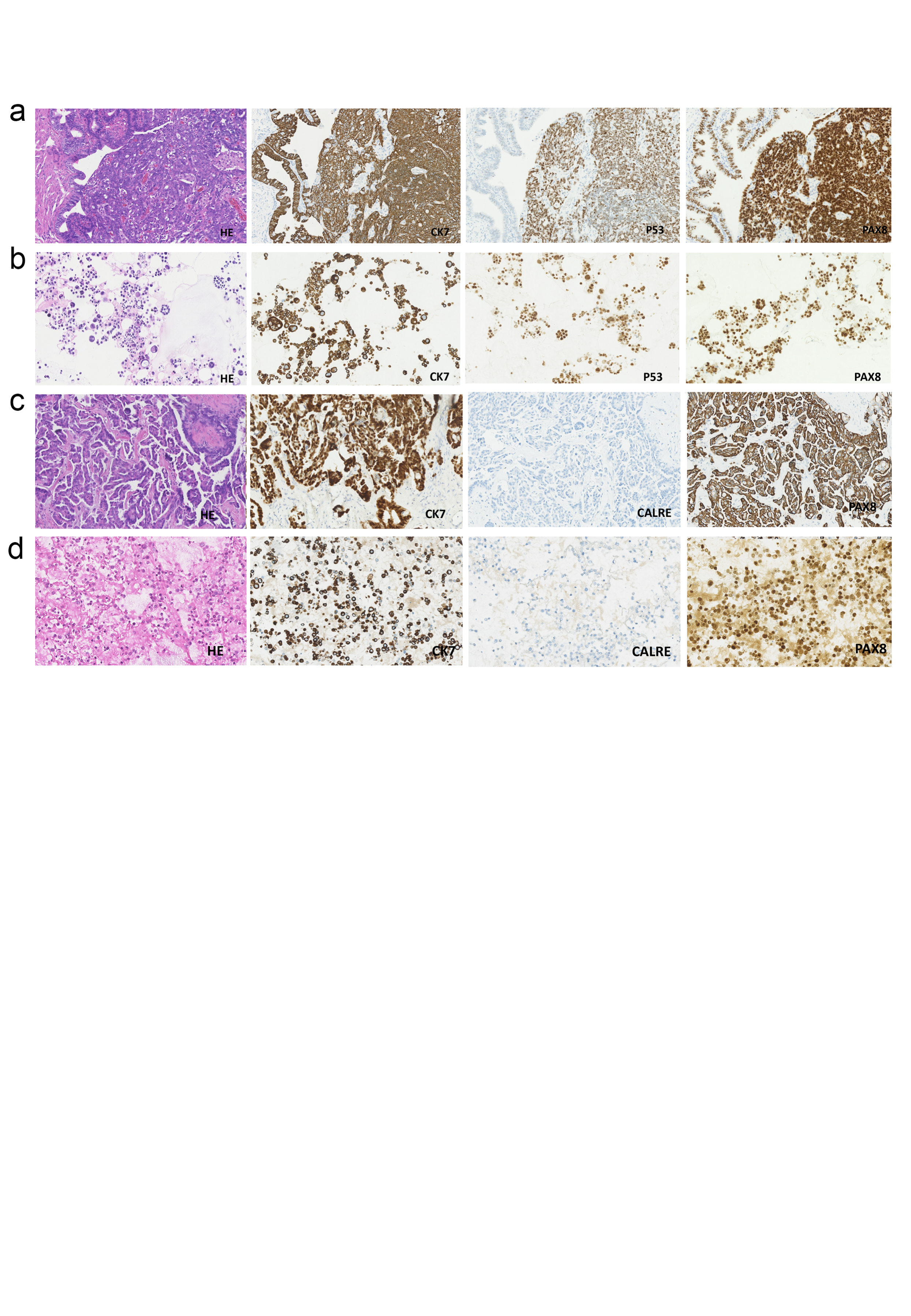

Supplement: Supplementary file 3 — Additional file 2: Fig. S2. (A) High-grade serous carcinoma showing strong and diffuse immunohistochemical expression of CK7, TP53, and PAX8. (B) Section from tumor-derived organoid day 11. The atypical cells show strong and diffuse immunohistochemical expression of CK7, TP53, and PAX, similarly to the primary tumor shown in (a). (C) Low-grade serous ovarian cancer shows a strong and diffuse immunohistochemical expression of CK7 and PAX8 and absent reaction for calretinin. (D) Tumor-derived organoids show atypical cells with strong and diffuse immunohistochemical expression of CK7 and PAX and an absence of reaction for calretinin, similarly to the primary low-grade serous ovarian cancer shown in (C). [file 12964_2022_991_MOESM3_ESM.tiff]

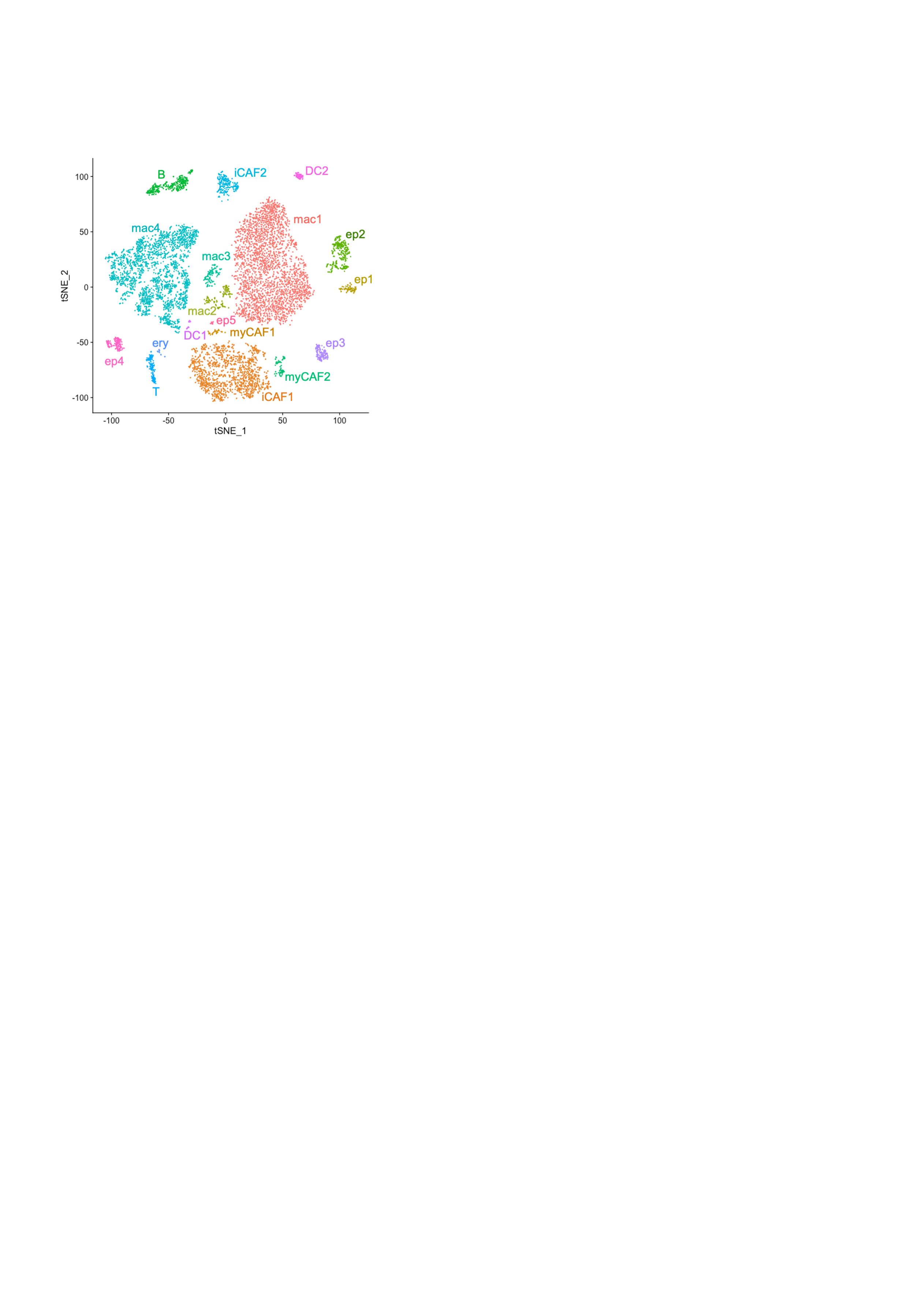

Supplement: Supplementary file 4 — Additional file 3: Fig. S3. Re-analysis based on single-cell RNA-seq data from malignant ascites of eight patients with advanced high-grade serous ovarian cancer described by Izar et al. [17]. The t-SNE shows 9.609 cells analyzed in 18 clusters (colors) that include ovarian cancer cells (Ep1-5), myofibroblastic cancer-associated fibroblasts (myCAF1-2), inflammatory cancer-associated fibroblasts (iCAF1-2), macrophages (mac1-4), dendritic cells (DC1-2), B cells (B), T cells (T), and erythrocytes (ery). [file 12964_2022_991_MOESM4_ESM.tiff]

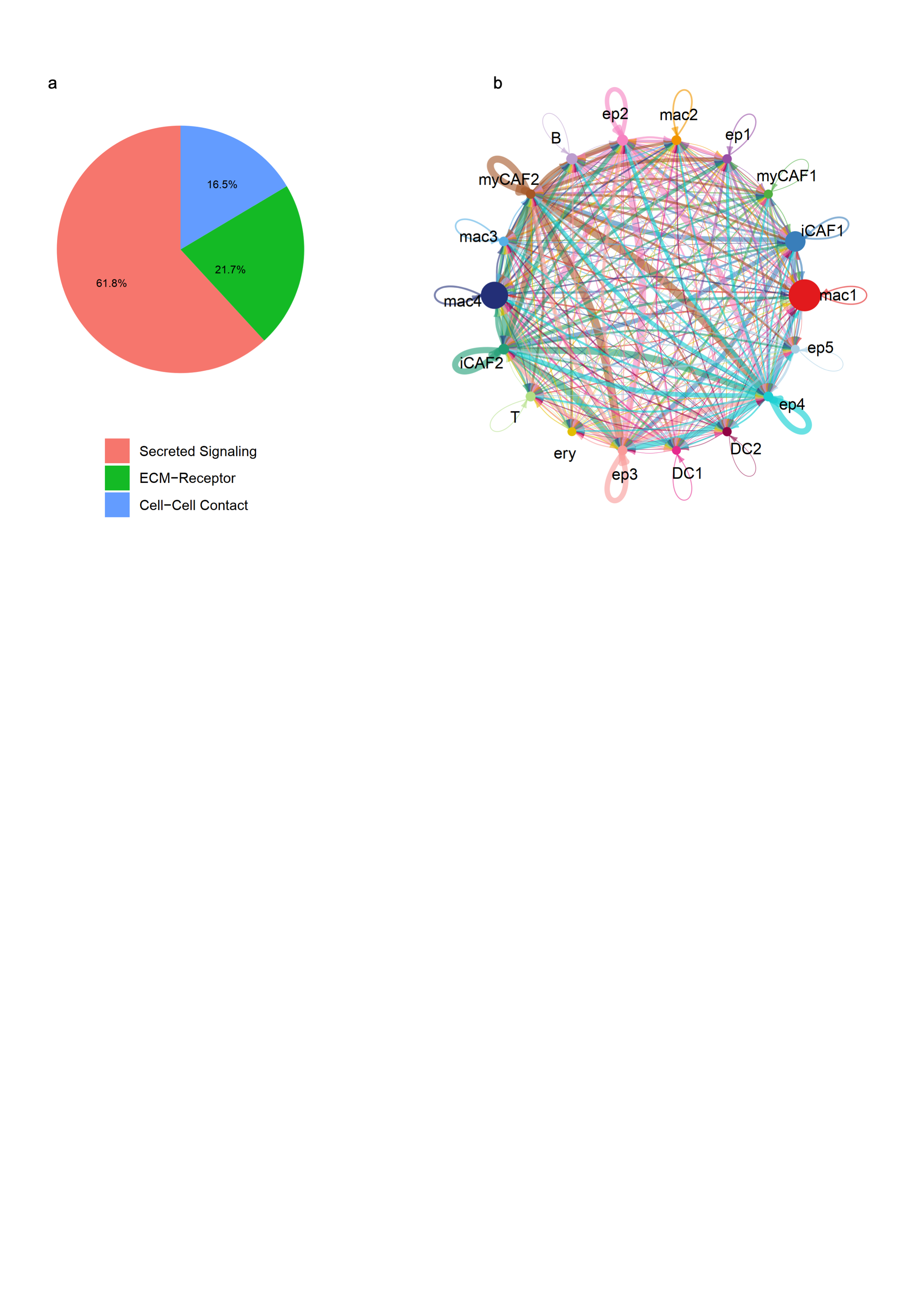

Supplement: Supplementary file 5 — Additional file 4: Fig. S4. (A) Literature-supported ligand-receptor interactions in humans available at the CellChatDB database (http://www.cellchat.org/) [35]. The 1,939 validated interactions included paracrine/autocrine signaling (61.8%), extracellular matrix (ECM) receptor interactions (21.7%) and cell-cell contact interactions (16.5%). (B) Circos plot showing the CellChatDB inferred cell-cell communication network based on single-cell RNA-Seq data across ovarian cancer cells (Ep1-5), myofibroblastic cancer-associated fibroblasts (myCAF1-2), inflammatory cancer-associated fibroblasts (iCAF1-2), macrophages (mac1-4), dendritic cells (DC1-2), B cells (B), T cells (T), and erythrocytes (ery) from ovarian cancer patients. [file 12964_2022_991_MOESM5_ESM.tiff]

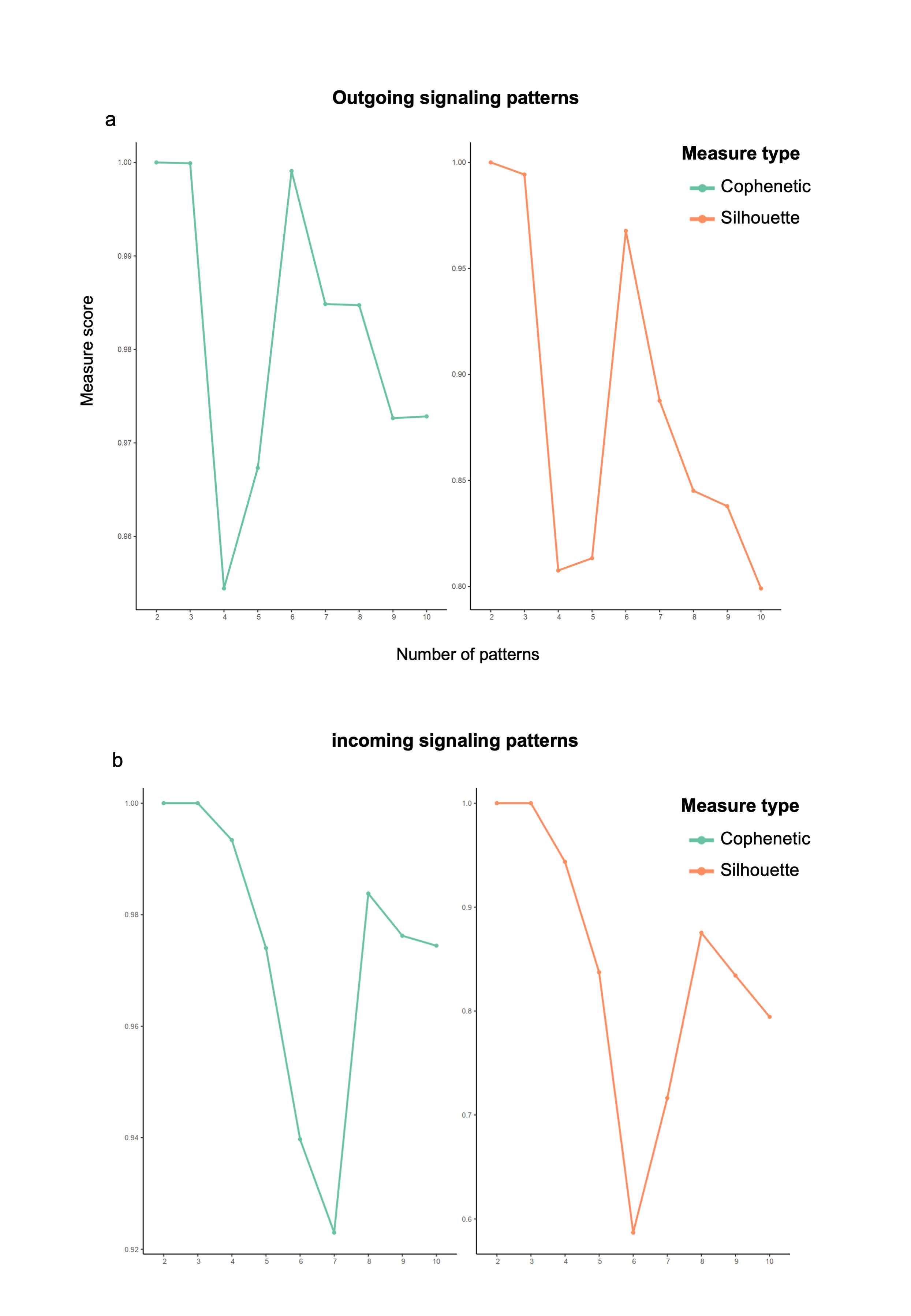

Supplement: Supplementary file 6 — Additional file 5: Fig. S5. Cophenetic and Silhouette metrics were used in CellChatDB [35] to identify the number of outgoing (A) and incoming (B) communication patterns that cell groups and pathways coordinate to function. Both Cophenetic and Silhouette values suggest three patterns, as indicated by the first sudden drop of the measured score in this number of patterns. [file 12964_2022_991_MOESM6_ESM.tiff]

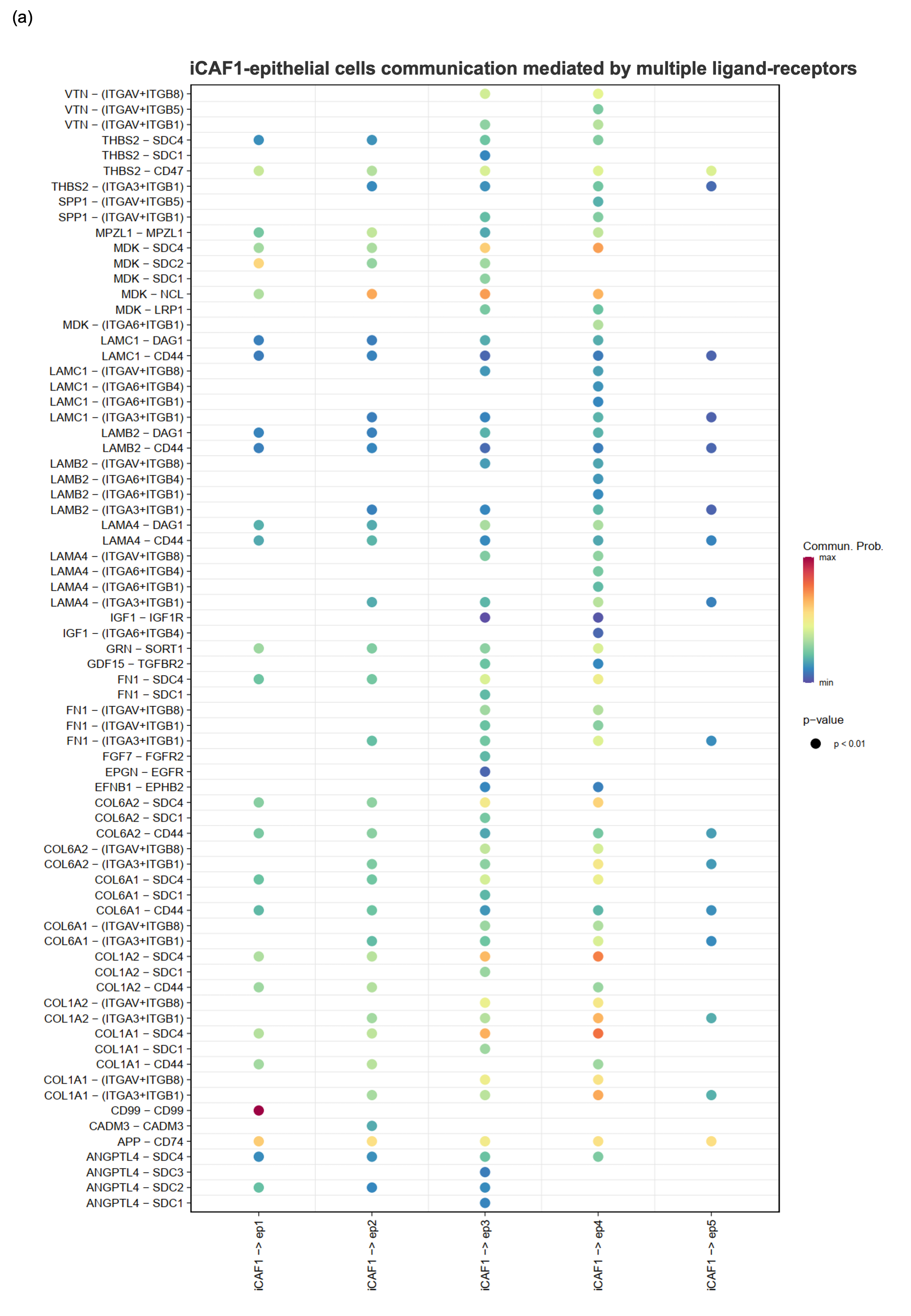

Supplement: Supplementary file 7 — Additional file 6: Fig. S6. Ligand-receptor interactions between cancer-associated fibroblasts and epithelial cells inferred by CellChatDB [35] using single-cell RNA-seq data [17] from malignant ascites of ovarian cancer patients. (A) Inflammatory cancer-associated fibroblasts 1 (iCAF1)-epithelial cells. [file 12964_2022_991_MOESM7_ESM.tiff]

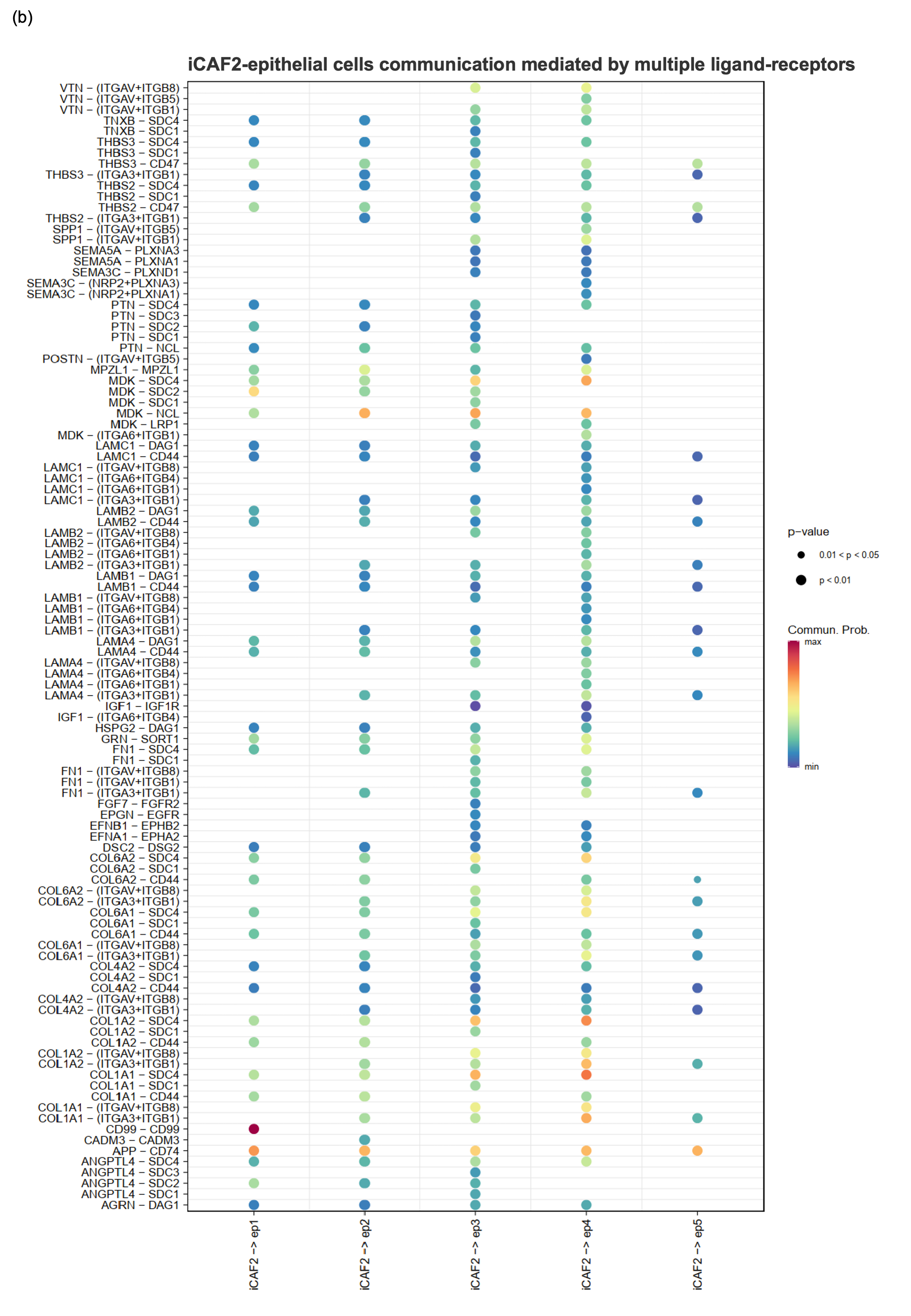

Supplement: Supplementary file 8 — Additional file 7: Fig. S6. (B) Inflammatory cancer-associated fibroblasts 2 (iCAF2)-epithelial cells. [file 12964_2022_991_MOESM8_ESM.tiff]

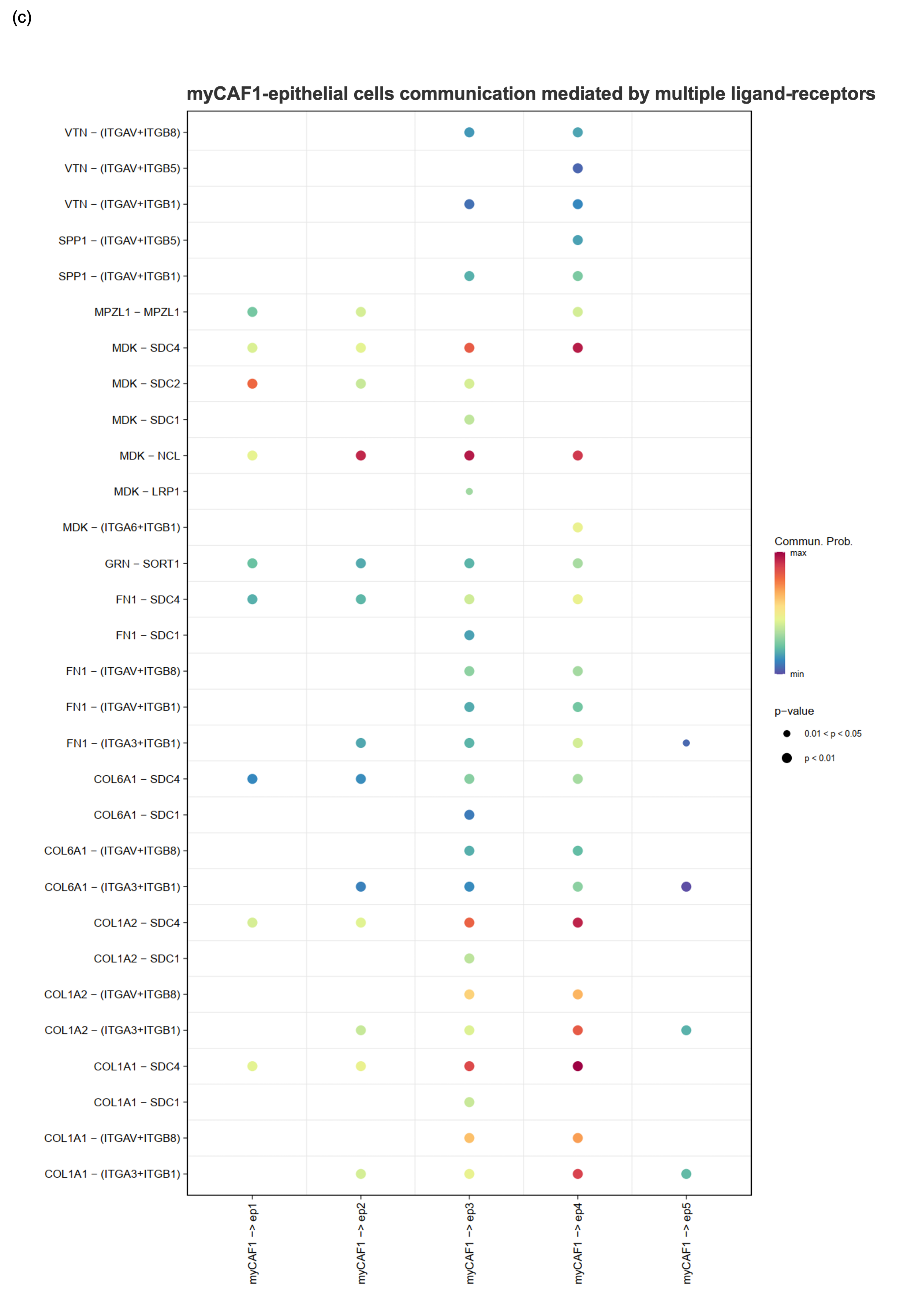

Supplement: Supplementary file 9 — Additional file 8: Fig. S6. (C) Myofibroblastic cancer-associated fibroblasts 1 (myCAF1)-epithelial cells. [file 12964_2022_991_MOESM9_ESM.tiff]

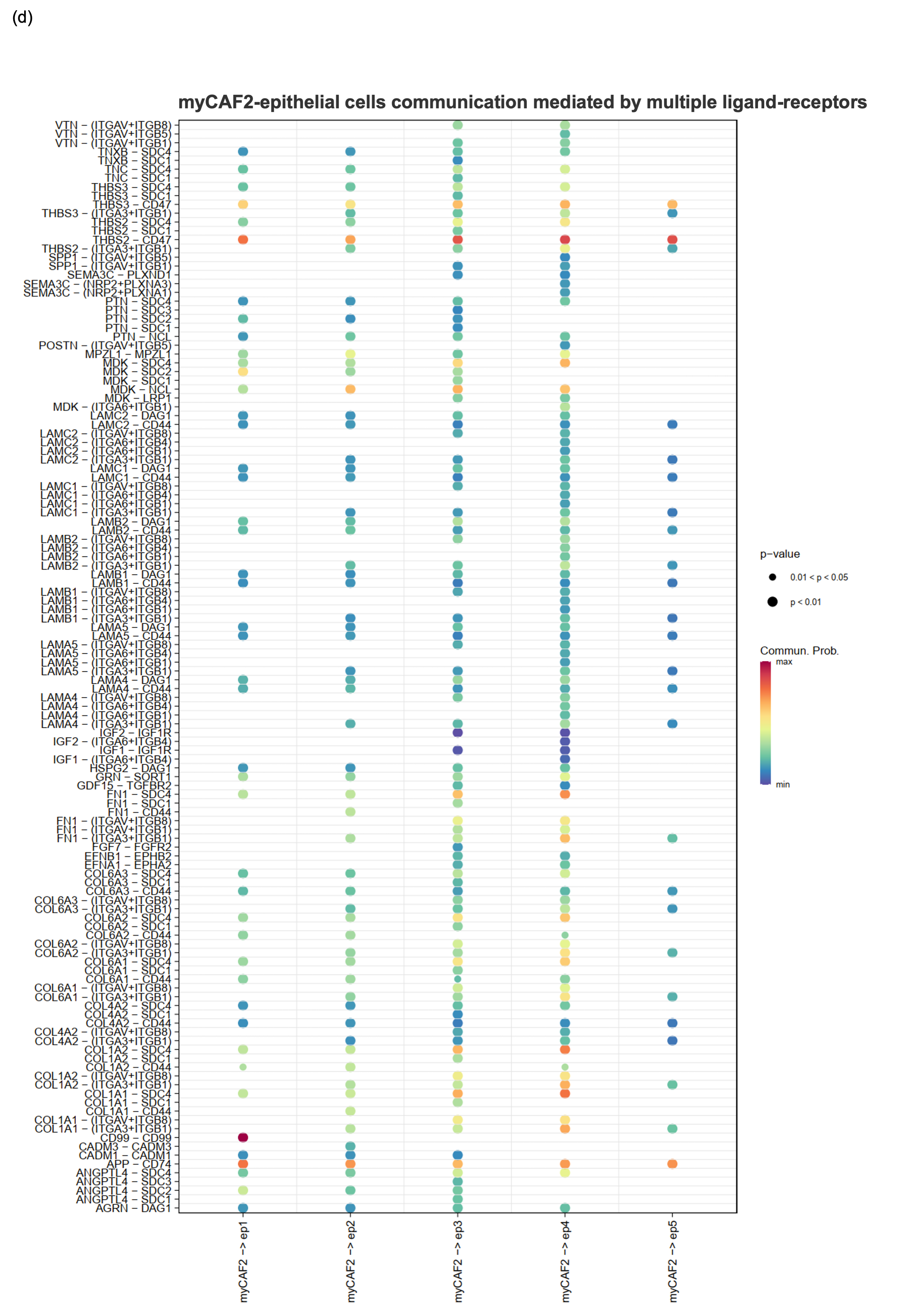

Supplement: Supplementary file 10 — Additional file 9: Fig. S6. (D) Myofibrobastic cancer-associated fibroblast 2 (myCAF2)-epithelial cells. [file 12964_2022_991_MOESM10_ESM.tiff]

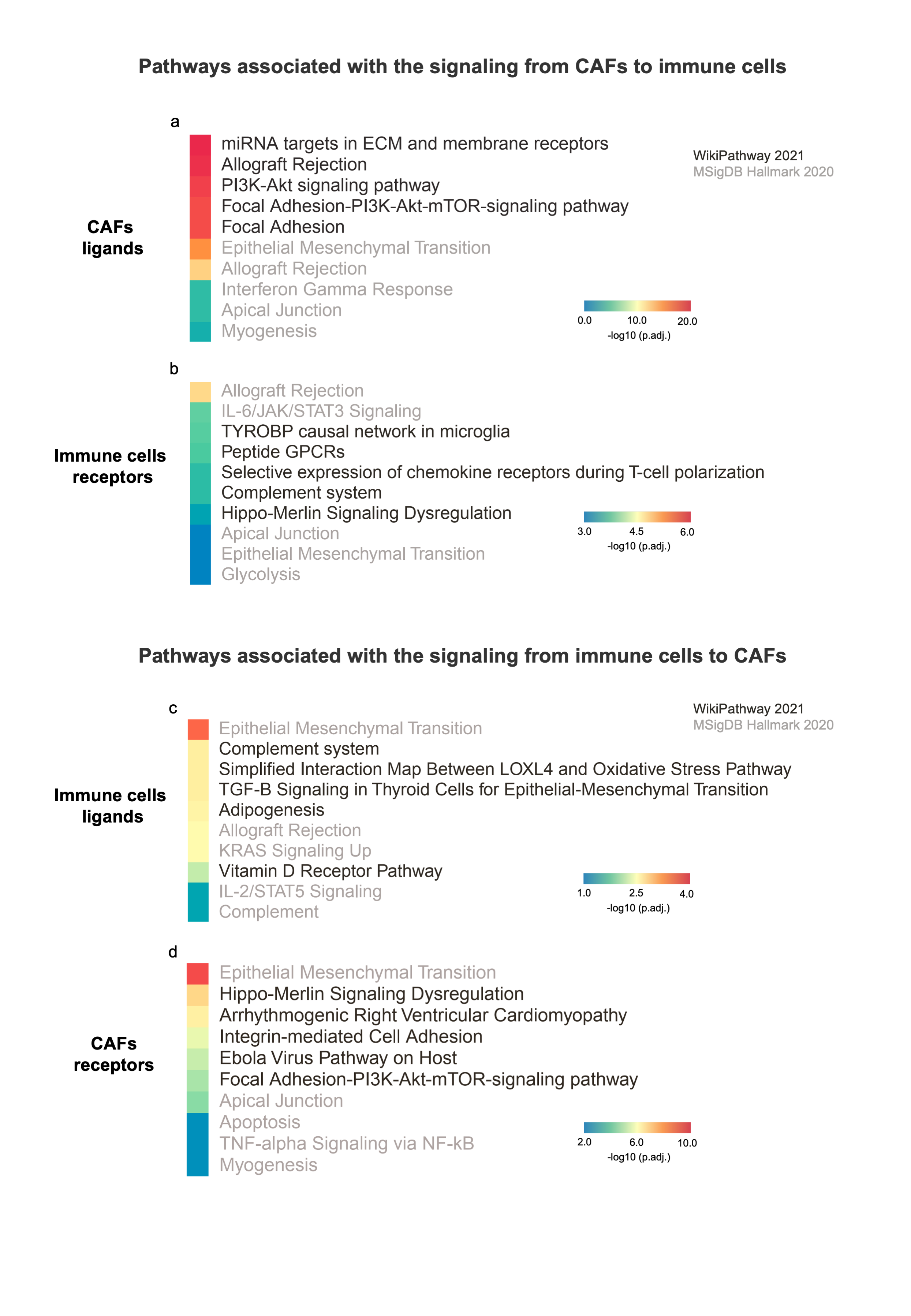

Supplement: Supplementary file 11 — Additional file 10: Fig. S7. Pathways associated with the signaling (ligands and receptors) from CAFs to immune cells (a and b) and from immune cells to CAFs (d and e). The top 10 pathway terms (lowest adjusted P-value) were selected from WikiPathways 2021 and MSigDB Hallmark 2020 libraries available in EnrichR [30, 31]. [file 12964_2022_991_MOESM11_ESM.tiff]

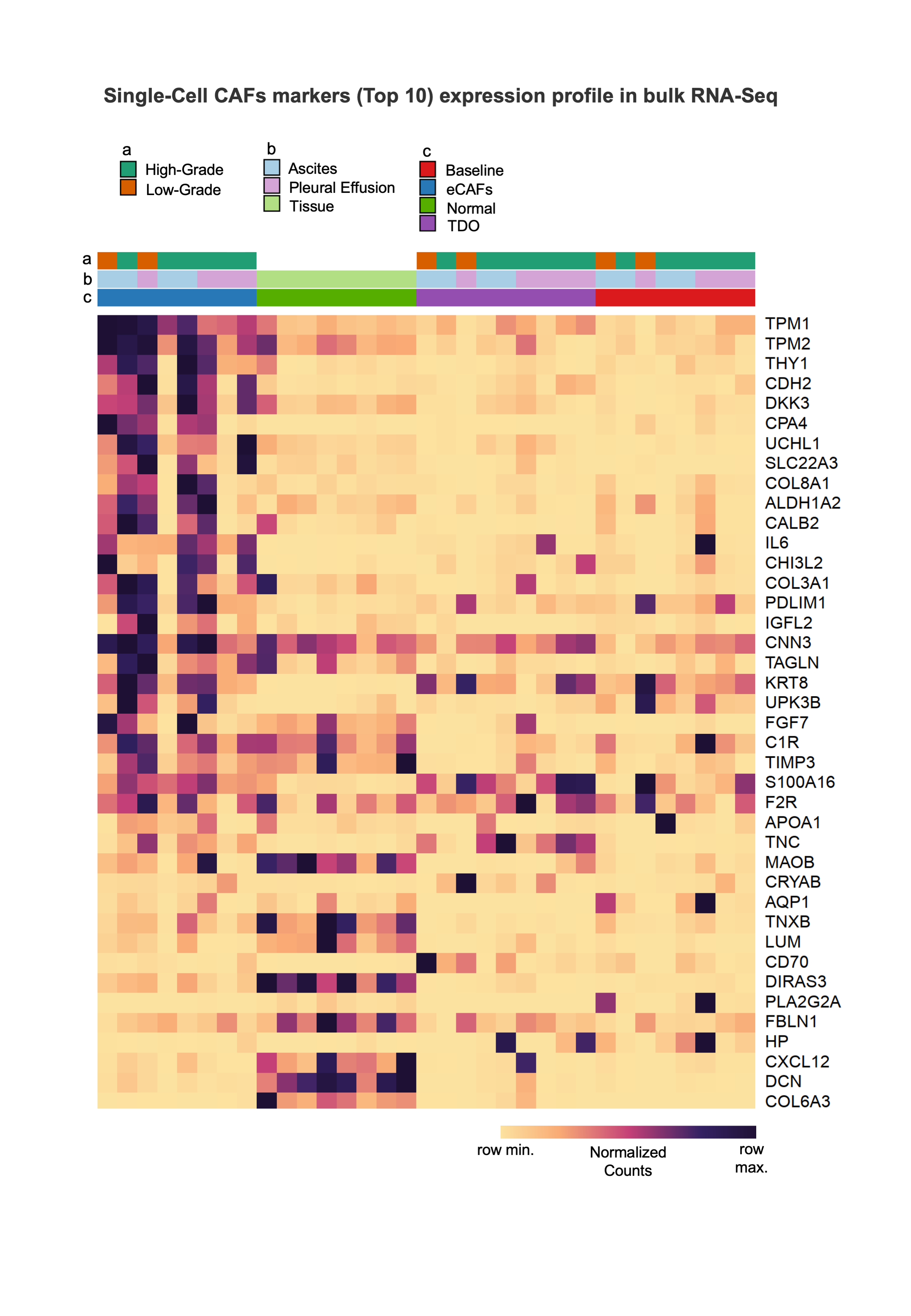

Supplement: Supplementary file 12 — Additional file 11: Fig. S8. Expression profile of 40 cancer-associated fibroblast markers (top 10 for each cluster from scRNA-Seq data) in ovarian bulk RNA-Seq. Genes were ordered based on a marker selection (signal to noise) that highlights the differences in the expression profile (normalized counts) between ovarian cancer-associated fibroblasts-enriched culture (eCAFs) and the other three conditions (Normal tissue, Normal; tumor-derived organoids, TDO; and baseline). (A) grade; (B) malignant effusions or normal tissue; and (C) experimental conditions. [file 12964_2022_991_MOESM12_ESM.tiff]

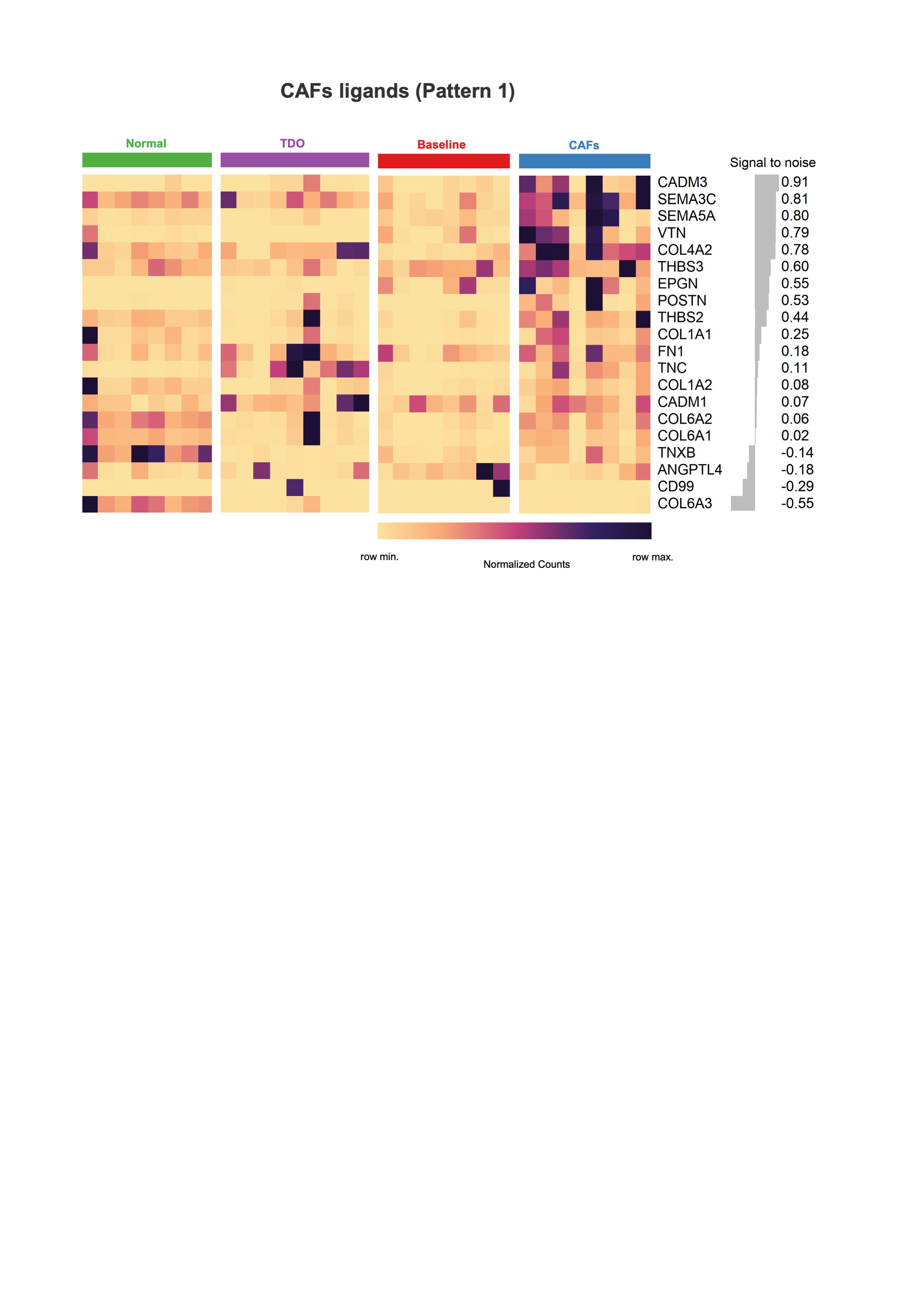

Supplement: Supplementary file 13 — Additional file 12: Fig. S9. Expression profiling of CAFs ligands (communication pattern 1) in ovarian bulk RNA-Seq. Genes were ordered based on a marker selection (signal to noise) that highlights the differences in the expression profile (normalized counts) between ovarian cancer-associated fibroblasts-enriched culture (eCAFs) and the other three conditions (Normal tissue, Normal; tumor-derived organoids, TDO; and baseline). [file 12964_2022_991_MOESM13_ESM.tiff]

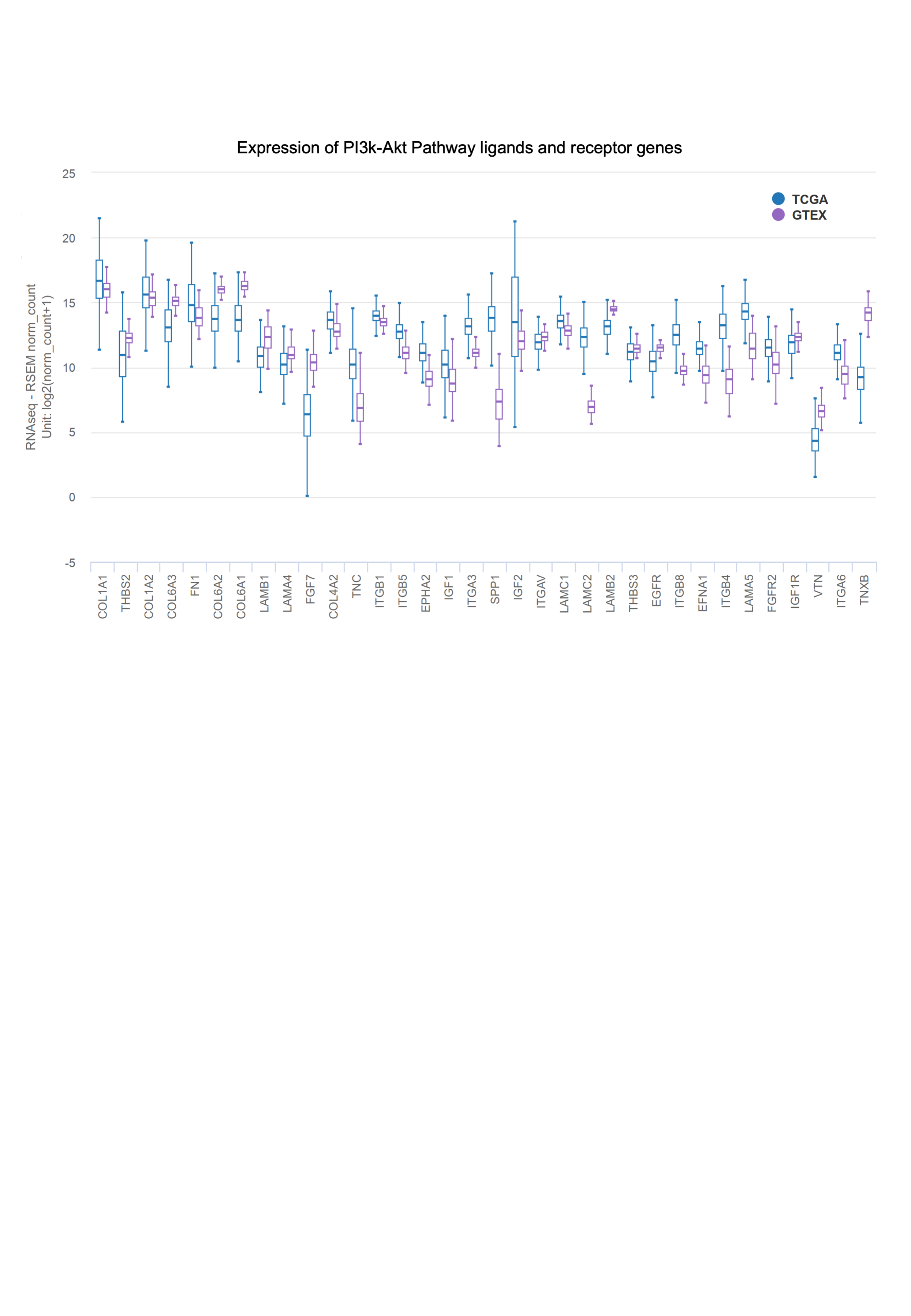

Supplement: Supplementary file 14 — Additional file 13: Fig. S10. Box plot showing the expression levels [log2(norm_count+1)] of ligands and receptors of the PI3K-AKT signaling pathway in ovarian cystadenocarcinoma (n = 419) of The Cancer Genome Atlas (TCGA) compared to normal ovarian tissues (n = 88) tissues of Genotype-Tissue Expression (GTEx). [file 12964_2022_991_MOESM14_ESM.tiff]
